# Cofilin activation in pancreatic acinar cells plays a pivotal convergent role for mediating CCK-stimulated enzyme secretion and growth

**DOI:** 10.3389/fphys.2023.1147572

**Published:** 2023-04-17

**Authors:** Irene Ramos-Alvarez, Lingaku Lee, Robert T. Jensen

**Affiliations:** ^1^ Digestive Diseases Branch, National Institute of Diabetes and Digestive and Kidney Diseases, National Institutes of Health, Bethesda, MD, United States; ^2^ National Kyushu Cancer Center, Department of Hepato-Biliary-Pancreatology, Fukuoka, Japan

**Keywords:** cofilin, CCK-8, PAK4, pancreatic acini, phosphatases, enzyme secretion, pancreatic growth

## Abstract

**Introduction:** The actin regulatory protein, cofilin plays a key signaling role in many cells for numerous cellular responses including in proliferation, development, motility, migration, secretion and growth. In the pancreas it is important in islet insulin secretion, growth of pancreatic cancer cells and in pancreatitis. However, there are no studies on its role or activation in pancreatic acinar cells.

**Methods:** To address this question, we studied the ability of CCK to activate cofilin in pancreatic acinar cells, AR42J cells and CCK_1_-R transfected Panc-1 cells, the signaling cascades involved and its effect on enzyme secretion and MAPK activation, a key mediator of pancreatic growth.

**Results:** CCK (0.3 and 100 nM), TPA, carbachol, Bombesin, secretin and VIP decreased phospho-cofilin (i.e., activate cofilin) and both phospho‐kinetic and inhibitor studies of cofilin, LIM kinase (LIMK) and Slingshot Protein Phosphatase (SSH1) demonstrated these conventional activators of cofilin were not involved. Serine phosphatases inhibitors (calyculin A and okadaic acid), however inhibited CCK/TPA-cofilin activation. Studies of various CCK‐activated signaling cascades showed activation of PKC/PKD, Src, PAK4, JNK, ROCK mediated cofilin activation, but not PI3K, p38, or MEK. Furthermore, using both siRNA and cofilin inhibitors, cofilin activation was shown to be essential for CCK-mediated enzyme secretion and MAPK activation.

**Conclusion:** These results support the conclusion that cofilin activation plays a pivotal convergent role for various cell signaling cascades in CCK mediated growth/enzyme secretion in pancreatic acini.

## Graphical Abstract

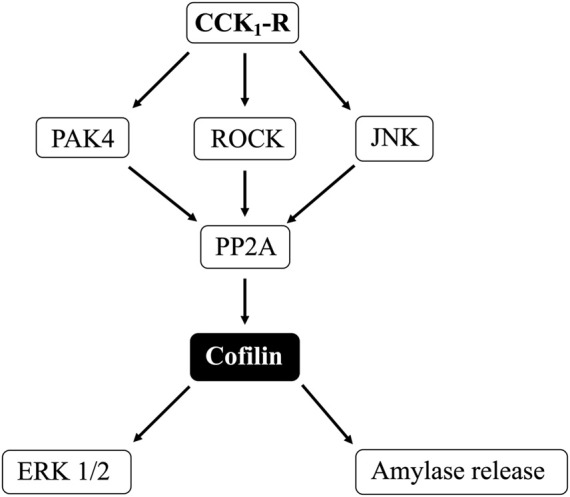

## 1 Introduction

Cofilin is an actin-binding protein of 21 kDA, which is a regulator of actin filament dynamics and depolymerization (Ohashi, 2015; Xu et al., 2021). Cofilin promotes the conversion of actin filaments by enhancing F-actin depolymerization and inhibiting G-actin polymerization, which are essential in actin filament dynamics (Ohashi, 2015; Xu et al., 2021). In numerous cells, cofilin activity is regulated by several molecular mechanisms including its inactivation by phosphorylation at the N-terminal Ser-3 and reactivation by dephosphorylation (Kaji et al., 2003; Xu et al., 2021). Cofilin activation is primarily regulated by alterations in activation of LIM kinase (LIMK) (deactivation) and Slingshot Protein Phosphatase (SSH1) (activation) (Ohashi, 2015; Xu et al., 2021). In numerous cells, cofilin is involved in a wide range of activities including the development of several tissues and organs, especially neural tissues; in cellular proliferation and migration; the establishment of cellular polarity; the dynamic regulation of organ morphology; mitosis; cytokinesis; secretion and growth (Won et al., 2008; Jayaram and Kowluru, 2012; Mizuno, 2013; Ohashi, 2015; Xu et al., 2021). In regard to pancreatic function, cofilin has been shown to be important in the regulation of insulin secretin by pancreatic islets (Jayaram and Kowluru, 2012), to be important in pancreatic tumor growth and metastatic behavior, similar to its role in other cancers (Xu et al., 2021), and in pancreatic acinar depolymerization/reorganization (Xu et al., 2021), which is important in the pathogenesis of pancreatitis (Yang et al., 2022). However, there is no information whether pancreatic acinar cell stimulants activate cofilin or its possible role in pancreatic acinar cell function, such as enzyme secretion or growth. However, even though little is known of the role of cofilin in pancreatic acinar cell function, activation of the actin cytoskeleton has been shown to be particularly important to modulate the secretory granule exocytosis in pancreatic and rat parotid acinar cells (Messenger et al., 2014). Moreover, the Rho family of small G proteins, RhoA and Rac1, also regulate pancreatic secretion through remodeling of the actin cytoskeleton in the pancreas (Williams et al., 2009). Therefore, because cofilin has been shown to be an essential actin regulatory protein that constitutively severs actin filaments, and thereby accelerates actin assembly and altering actin activity in other tissues (Ohashi, 2015; Xu et al., 2021), one would suspect that cofilin activation could play a major role in pancreatic secretion, and possibly growth.

To address this question, we examined the effect of various hormones/neurotransmitters known to alter pancreatic acinar activity/function to activate cofilin in acinar cells. We extended these studies by examining in detail the ability of cholecystokinin, a physiological regulator of pancreatic secretion/growth (Dufresne et al., 2006), on pancreatic acinar cofilin activation, on cofilin’s possible role in pancreatic growth/enzyme secretion and elucidated the signaling cascades involved.

## 2 Materials and methods

### 2.1 Materials

Male Sprague–Dawley rats (100–120 g) were obtained from the Small Animals Section, Veterinary Resources Branch, National Institutes of Health (NIH), Bethesda, MD. Phospho-PAK4(Ser474)/PAK5(Ser602)/PAK6(Ser560) was from GeneTex (Irvine, CA). Phospho-pS3 cofilin, cofilin, Phospho-pT508 LIMK, PP1, protein phosphatase 2 (PP2), α/β-Tubulin, Phospho-p44/42 MAPKs (Erk1/2) (Thr202/Tyr204) and p44/42 MAPK (Erk1/2) were from Cell Signaling Technology, Inc. (Beverly, MA). Stabilized goat anti-rabbit IgG peroxidase conjugate was from Pierce Biotechnology, Inc. (Rockford, IL). Sennoside A (SE), PAK4 (P-21), anti-goat-HRP-conjugate antibodies, calyculin A and cofilin antibody were from Santa Cruz Biotechnology, Inc. (Dallas, TX). Phospho-pS978 Slingshot-1 (pS978-SSH1) and SSH1 antibodies were from ECM Bioscience (Versailles, KY). Tris/HCl pH 8.0 and 7.5 were from Mediatech, Inc. (Herndon, VA). 2-mercaptoethanol, protein assay solution, sodium lauryl sulfate (SDS) and Tris/Glycine/SDS (10×) were from Bio-Rad Laboratories (Hercules, CA). MgCl_2_, CaCl_2_, Tris/HCl 1 M pH 7.5 and Tris/Glycine buffer (10X) were from Quality Biological, Inc. (Gaithersburg, MD). Dulbecco’s minimum-essential medium (DMEM), RPMI 1640, fetal bovine serum (FBS), Lipofectamine™ RNAiMAX, OPTI-MEM, trypsin-EDTA, penicillin/streptomycin, amino acids 100X, 4%–20% Tris–Glycine gels and ethidium bromide solution were from Invitrogen (Carlsbad, CA). 12-O-tetradecanoylphobol-13-acetate (TPA), L-glutamic acid, glucose, fumaric acid, pyruvic acid, trypsin inhibitor, HEPES, TWEEN^®^ 20, Triton X-100, phenylmethanesulfonylfluoride (PMSF), ethylenediaminetetraacetic acid (EDTA), ethylene glycol tetra-acetic acid (EGTA), sucrose, sodium-orthovanadate, sodium azide, albumin standard and Super Signal West (Pico, Dura) chemiluminescent substrate were from Pierce (Rockford, IL). Protease inhibitor tablets were from Roche (Basel, Switzerland). Purified collagenase (type CLSPA) was from Worthington Biochemicals (Freehold, NJ). Nitrocellulose membranes were from Schleicher and Schuell Bioscience, Inc. (Keene, NH). NaCl, KCl and NaH_2_PO_4_ were from Mallinckrodt (Paris, KY). Non-Fat milk Ominlok was purchased from AmericanBio (Natick, MA). PF-3758309 was from APRxBIO (Houston, TX). Phadebas^®^ Amylase test was from Magle Life Sciences (Cambridge, MA). N2-(3-methoxyphenyl)-N4-[(oxolan-2-yl)methyl]quinazoline-2,4-diamine (LCH-7749944) was from Moltport Gets molecules delivered (Riga, LV). (Z)-4-((4-((4-oxo-2-thioxo-3-(o-tolyl)thiazolidin-5-ylidene)methyl)phenoxy)methyl)benzoic acid (Slingshot inhibitor D3) was from AOBIOUS (Gloucester, MA). COOH-terminal octapeptide of cholecystokinin (CCK-8) and A71378 were from Bachem Bioscience Inc. (King of Prussia, PA). Okadaic acid was from Calbiochem (Gibbstown, NJ). LIMKi3 and SR7826 were from TOCRIS bioscience (Bristol, UK). Phospho-pY307 PP2 and SuperSignal™ West Dura Extended Duration Substrate were from Thermo-Fisher (Waltham, MA). GFX109203X (GFX), kbNB142-70 (kbNB), PP2 (Src inhibitor), FK-5046, Cytochalasin D, SP600125, Dexamethasone, AR42J cells and Sample Buffer Laemlii 2x concentrate were from Sigma-Aldrich (St. Louis, MO). Paclitaxel, U0126, Wortmannin and LY294002 were from Millipore (Burlington, MA). SB202190 was from APExBIO (Boston, MA). Y-27632 was from STEMCELL Technologies (Seattle, WA).

### 2.2 Methods

#### 2.2.1 Cell culture

CCK_1_ receptor transfected Panc-1 (CCK_1_-R/Panc-1) cells (Berna et al., 2009) and AR42J cells, were cultured in DMEM supplemented with 10% FBS and 1% penicillin/streptomycin and in RPMI supplemented with 10% FBS and 1% penicillin/streptomycin, respectively; and split 1:10 weekly with trypsin/EDTA, after washing in PBS. Finally, cells were seeded in 6-well plate and serum starved overnight, until they were 80% confluent. Cells were incubated at 37°C in 5% CO_2_/95% air. AR42J cells were treated with 100 nM dexamethasone for 72°h, which stimulates their differentiation into exocrine cells (Logsdon, 1986).

#### 2.2.2 Pancreatic acini preparation, stimulation and inhibition experiments

Pancreatic acini preparations were obtained by collagenase digestion as previously described (Bissonnette et al., 1984; Sato et al., 1989; Ramos-Alvarez and Jensen, 2018). After collagenase digestion, dispersed acini were preincubated with different inhibitors in standard incubation solution (Ramos-Alvarez and Jensen, 2018; Ramos-Ãlvarez et al., 2020) for 3 h at 37°C under conditions specific for PKD/PKC, Src, PI3K, MAPKs, SSH1, LIMK, cofilin, other phosphatases and PAK4 inhibitors, as described previously (Ramos-Alvarez and Jensen, 2018; Ramos-Ãlvarez et al., 2020). Isolated acini and CCK_1_-R/Panc-1 cells (M&M 2.2.1) were also preincubated with two SSH1 inhibitors, D3 (25 μM, 1 h) (Li et al., 2015) or SE (50 μM, 3 h for acini; 24 h for CCK_1_-R/Panc-1 cells) (Lee et al., 2017). After preincubation, 1 mL aliquots of dispersed acini were incubated at 37°C for 3–15 min with CCK-8 (0.3 nM or 100 nM), for 5–15 min with TPA (1 µM) or without stimulants, used as control. Cells were lysed in lysis buffer (50 mM Tris/HCl pH 7.5, 150 mM NaCl, 1% Triton X-100, 1% deoxycholate, 0.1% sodium azide, 1 mM EGTA, 0.4 mM EDTA, 0.2 mM sodium orthovanadate, 1 mM PMSF, and one protease inhibitor tablet per 10 mL). After sonication, lysates were centrifuged at 10,000×*g* for 15 min at 4°C and protein concentration was measured using the Bio-Rad protein assay reagent. Finally, cells were processed as below for Western blotting.

To select appropriate concentrations, we performed preliminary time courses (1–3 h) and dose-response curves (1–50 μM and 1–50 nM) (data not shown) with the different inhibitors. These results demonstrated that the maximal inhibitory effect was seen after 3 h (except for D3, Wortmannin, LY294002, SB202190 and U0126 at 1 h) of preincubation with concentrations of; **(A)** SSH1 Inhibitors, D3 at 25 µM and SE at 50 µM (Li et al., 2015; Lee et al., 2017); **(B)** LIMK inhibitors, LIMKi3 at 10 µM and SR7826 at 10 μM; **(C)** Phosphatase inhibitors, calyculin A at 1 nM and okadaic acid at 10 nM; **(D)** PKC/PKD, Src inhibitors, GFX at 5 μM, kbNB at 0.1 µM and PP2A at 10 μM, respectively (Ramos-Alvarez and Jensen, 2018), and calcineurin inhibitor, FK-506 at 10 μM; **(E)** PAK4 inhibitor, PF-3758309 (0.1 nM) and LCH-7749944 (30 µM) (Ramos-Alvarez and Jensen, 2018; Ramos-Alvarez et al., 2019); **(F)** Cofilin inhibitors, Cytochalasin D at 10 μM, and Paclitaxel at 5 μM; **(G)** PI3K inhibitors, Wortmannin at 1 µM and LY294002 at 100 µM (Ramos-Alvarez and Jensen, 2018); **(H)** p38 inhibitor, SB202190 at 10 µM (Ramos-Alvarez and Jensen, 2018); **(I)** MEK inhibitor, U0126 at 10 μM; **(J)** JNK inhibitor, SP600125 at 20 µM and **(K)** ROCK inhibitor, Y-27632 at 10 µM (Rak et al., 2014). Both concentrations of CCK-8 were used because CCK-8 is reported to have different responses with physiological and supraphysiological concentrations contributing to physiological and pathophysiological processes such as pancreatitis (Dufresne et al., 2006).

#### 2.2.3 Western blotting, immunoprecipitation and co-immunoprecipitation

Western blotting and immunoprecipitation were performed as described previously (Ramos-Alvarez and Jensen, 2018). Whole cell lysates were subjected to SDS-PAGE using 4%–20% Tris–Glycine gels. After electrophoresis, proteins (10–50 µg) were transferred to nitrocellulose membranes; blocked in blocking buffer (50 mM Tris/HCl pH 8.0, 2 mM CaCl_2_, 80 mM NaCl, 0.05% Tween^®^ 20, 5% nonfat dry milk) at room temperature for 1 h, and then, incubated with primary antibody overnight at 4°C under constant agitation at antibody dilutions suggested by the supplier (1:1,000); washed twice in blocking buffer for 4 min and then incubated with HRP-conjugated secondary antibody (anti-mouse, anti-rabbit, anti-goat, Dilution: 1:10,000), according to the species of the first antibody for 1 h at room temperature under constant agitation. Finally, membranes were then washed again twice in blocking buffer for 4 min, twice in washing buffer for 4 min, incubated for 4 min with chemiluminescence detection reagents. The intensity of the protein bands was measured using GeneTools software from Syngene, which were assessed in the linear detection range.

For co-immunoprecipitation, 600 μg of protein was incubated with 15 μL of the anti-cofilin antibody (Santa Cruz Biotechnology, Inc. Dallas, TX) and 25 μL of protein A/G agarose beads (Thermo-Fisher Scientific. Waltham, MA) overnight at 4°C under agitation. Samples were washed three times with lysis buffer, re-suspended in 12.5 μL of Sample Buffer, Laemlii 2x concentrate and boiled for 5 min before western blotting.

#### 2.2.4 siRNA assay

For siRNA-mediated knockdown, 5 × 10^5^ CCK_1_-R/Panc-1 or AR42J cells were seeded into 6-well plates and incubated for 24 h. Cells were then transfected with 10 nM of siRNA against human SSH1 siRNA against human *cofilin* (Dharmacon. Lafayette, CO) in Opti-MEM medium for 48 h using Lipofectamine according to the manufacture’s instruction. CCK_1_-R/Panc-1 or AR42J cells were transfected with 10 nM of or non-targeting control-siRNA (Dharmacon. Lafayette, CO) to rule out the possibility that siRNA against SSH1 or cofilin had an effect in the siRNA experiment (Baum et al., 2010).

#### 2.2.5 Amylase release

Amylase release was performed as described previously (Sato et al., 1989; Ramos-Alvarez and Jensen, 2018). Amylase activity was determined after 30 min incubation using the Phadebas reagent and was expressed as percentage of the total cellular amylase released into the extracellular medium during the incubation (Ramos-Alvarez and Jensen, 2018).

#### 2.2.6 Statistical analysis

All experiments were performed at least 3 times. Data are presented as mean ± SEM and were analyzed with the non-parametric Kruskal–Wallis analysis (data was not normally distributed) and the Dunn’s multiple comparison test using the GraphPad Prism 8.0 software. *P* values < 0.05 were considered significant.

## 3 Results

### 3.1 Stimulation of various secretagogues and dose-response effect of CCK-8 and CCK-JMV on cofilin activation

**FIGURE 1:**
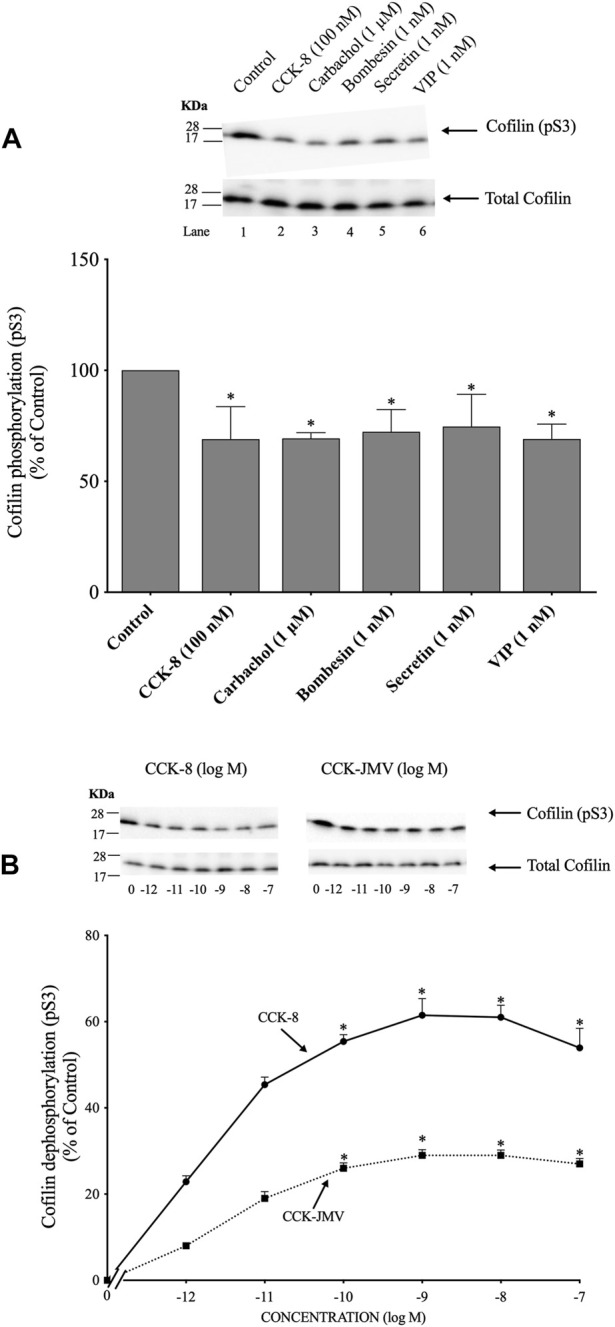
Ability of various pancreatic acinar secretagogues and dose-response effect of cholecystokinin (CCK)-8 and CCK-JMV to stimulate cofilin in rat pancreatic acini. **(A)** Isolated pancreatic acini were incubated in the absence or presence of CCK-8 (100 nM), carbachol (1 μM), bombesin (1 nM), secretin (1 nM) or VIP (1 nM) for 1 min and then lysed. **(B)** Isolated pancreatic acini were incubated in the absence or presence of CCK-8 and CCK-JMV (at the indicate concentrations) for 3 min and then lysed. Western blots were analyzed using anti-pS3 cofilin, which mediates the activation state of cofilin. Bands were visualized using chemiluminescence and quantified by densitometry. *Top*: Results of a representative blot of three independent experiments are shown. *Bottom*: Means ± S.E. of at least 3 independent experiments. Results are expressed as % of basal stimulation of the control group. *, *p* < 0.05 compared to the control group.

(Figure 1) To determine if cofilin was activated by various known pancreatic acinar cell activators (Jensen, 1994; Chandra and Liddle, 2007; Williams, 2019), rat pancreatic acini were incubated with and without CCK-8, carbachol, bombesin, secretin and VIP. As an initial general measurement of cofilin activation, we first analyzed the phosphorylation of cofilin at pS3 in response to various pancreatic stimulants. Numerous studies in other tissues with various stimulants have shown that cofilin activity is regulated by several different signaling cascades converging on the regulation of phosphorylation at the N-terminal Ser-3 of cofilin, with inactivated occurring with its phosphorylation and activated occurring with its dephosphorylation (Kaji et al., 2003; Mizuno, 2013; Ohashi, 2015; Xu et al., 2021). The assessment of this phosphorylation site has been widely used to assess activation of cofilin (Kaji et al., 2003; Mizuno, 2013; Ohashi, 2015; Xu et al., 2021). All the secretagogues tested in this study rapidly decreased cofilin phosphorylation (i.e., activated cofilin) (Figure 1A, Lanes 2–6).

Because CCK is a major physiological regulator of pancreatic acinar cell function (Jensen, 1994; Chandra and Liddle, 2007; Williams, 2019), and is also important in pathophysiological models of pancreatitis (Gukovskaya et al., 2002), we subsequently concentrated our studies on CCK’s effect on activation of cofilin and the signaling cascades involved. CCK-8 produced a detectible decrease in cofilin phosphorylation at 0.001 nM (Figure 1B), maximal dephosphorylation (i.e., activation) at 1 nM CCK-8 (62%), and a half-maximal effect (EC_50_) at 0.0018 ± 0.0001 nM (Figure 1B). In pancreatic acini, the CCK_1_ receptor exists in two different activation states, a low and a high affinity state, which can activate different cell signaling cascades (Stark et al., 1989; Berna et al., 2007; Ramos-Alvarez and Jensen, 2018; Williams, 2019). To determine the participation of each affinity state in the activation of cofilin by CCK-8, acini were incubated with CCK-JMV, an agonist of the CCK_1_ high affinity state and an antagonist of the low affinity CCK_1_ receptor state (Sato et al., 1989; Stark et al., 1989; Berna et al., 2007). CCK-JMV decreased cofilin phosphorylation (i.e., increasing activation) with concentrations from 0.1 nM to 100 nM (Figure 3B) with an EC_50_ of 0.005 ± 0.0001 nM (Figure 1B). These results demonstrate that 58% of the CCK-8 stimulation of cofilin activation is mediated by the high-affinity state CCK_1_ receptor and 42% by activation of the low affinity CCK_1_ receptor state (Figure 1B).

### 3.2 Time course of CCK/TPA stimulated activation/inactivation of LIMK, cofilin and SSH1

**FIGURE 2:**
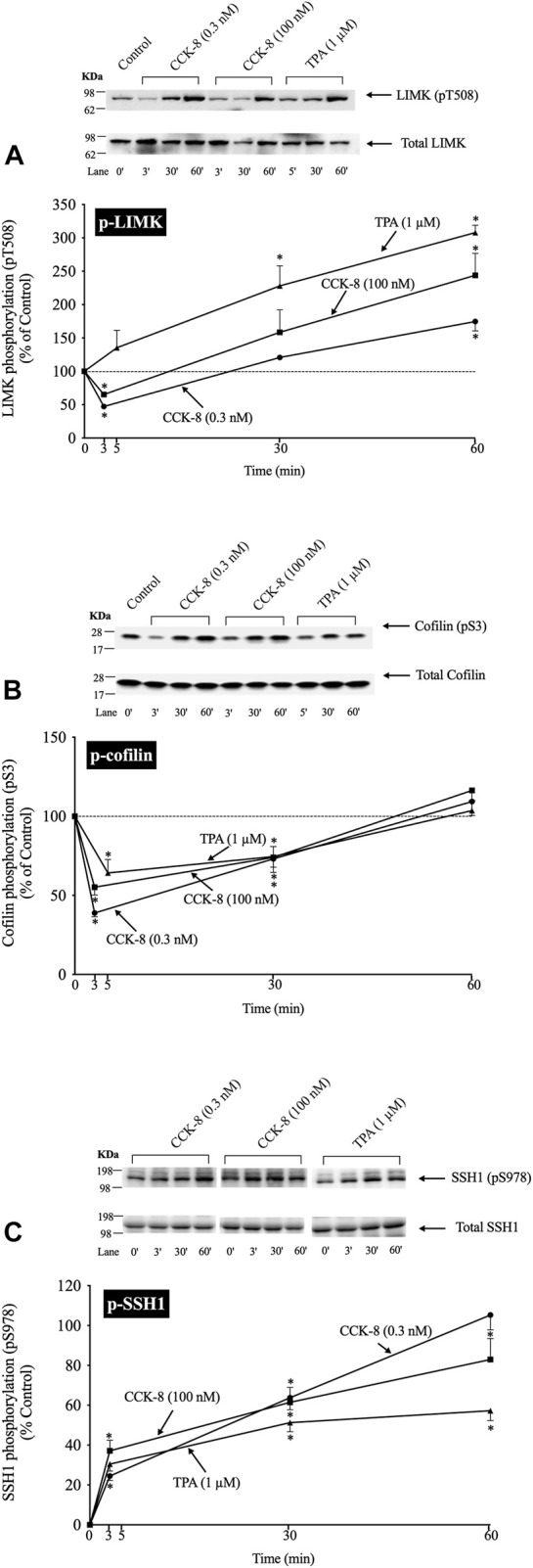
Time course of phosphorylation of Lim kinase (LIMK) **(A)**, cofilin **(B)** and Slingshot 1 phosphatase (SSH1) **(C)** induced by CCK-8 and TPA. Isolated pancreatic acini were incubated with either no addition (control), CCK-8 (0.3 and 100 nM) or TPA (1 µM) for the indicated periods of time and then lysed. Western blots were analyzed using anti-pT508 LIMK, anti-pS3 cofilin and anti-pS978 SSH1, which mediate the activation states of LIMK, cofilin and SSH1. Bands were visualized using chemiluminescence and quantified by densitometry. *Top*: Results of a representative blot of three independent experiments are shown. *Bottom*: Means ± S.E. of at least 3 independent experiments. Results are expressed as % of basal stimulation of the control group. *, *p* < 0.05 compared to the control group.

(Figure 2) In a number of previous studies in nonpancreatic tissues report (Spratley et al., 2011; Doppler et al., 2014) the activation status of cofilin is primarily regulated by alterations in activation of LIMK and the phosphatase slingshot homolog 1 (SSH1) (Ohashi, 2015; Xu et al., 2021). The phosphorylation of LIMK at pT508 results in its activation (Xu et al., 2021) which subsequently phosphorylates cofilin at pS3, leading to its inactivation (Xu et al., 2021). In contrast, the dephosphorylation of SSH1 at pS978 results in its activation leading to dephosphorylation of cofilin at pS3, resulting in activation of cofilin (Xu et al., 2021).

To examine the ability of CCK-8 to regulate the cofilin pathway, we first studied the time-dependent ability of CCK-8 (0.3 or 100 nM) and TPA, which directly activates PKC, one of the principal cascades activated by CCK (Dufresne et al., 2006), to regulate the LIMK/cofilin pathway and SSH1. The ability of CCK-8/TPA to alter the activation of cofilin, LIMK and SSH1 was assessed by determinizing the phosphorylation status of specific sites which have been shown to control their activity: pS3 cofilin, (Kaji et al., 2003; Xu et al., 2021); pT508 LIMK, (Li et al., 2010); and pS978 SSH1, (Eiseler et al., 2009).

With p-LIMK, CCK-8 (0.3 or 100 nM) demonstrated a biphasic time course, with a rapid dephosphorylation (i.e., deactivation) at 3 min (35%–53%), returning to the basal levels at 30 min, and significantly increasing phosphorylation by 60 min (175%–244%, Figure 2A, Lane 1 vs. 2–7). TPA did not cause LIMK dephosphorylation and instead increased the phosphorylation levels of LIMK (i.e., increasing activation) at 30–60 min (228%–309%, Figure 2A, Lane 1 vs. 8–10). With cofilin, both CCK-8 (0.3 or 100 nM) and TPA caused rapid maximal dephosphorylation (i.e., activation) at 3 min (39%–64%), and then with time the phosphorylation returned to the original basal level at 60 min (Figure 2B). For SSH1, both CCK-8 (0.3 or 100 nM) and TPA produced a rapid increased in SSH1 phosphorylation (i.e., deactivation) which was first detected at 3 min with a maximum increment after 30 min (164%, 162% and 151%, respectively, Figure 2C, Lane 1 vs. 3; 5 vs. 7 and 9 vs. 11) and it was still present after 60 min (Figure 2C).

These results demonstrate both CCK-8 (at both 0.3 and 100 nM) and TPA can regulate phosphorylation at the primary sites shown to be involved in the regulation of the activation of cofilin, LIMK and SSH1, in numerous tissues by different stimuli (Eiseler et al., 2009; Xu et al., 2021). Furthermore, these results demonstrate that both concentrations of CCK-8 and TPA stimulate rapid activation of cofilin (i.e., dephosphorylation of pS3), whereas with LIM kinase, CCK stimulated deactivation initially (dephosphorylation of pT508 LIMK), followed by reactivation (increased pT508 LIMK). TPA, in contrast, only stimulated activation of LIM kinase, whereas TPA and CCK deactivated SSH1 (increased pS978 phosphorylation). To rule out the possibility SSH1 was activated at very short incubation time points that was missed with these longer incubation times, we did additional studies at 0.5 min and 1.5 min incubation times and no dephosphorylation of SSH1 (activation) was seen (data not shown) supporting the conclusion that CCK-8/TPA were not activating SSH1. Because the highest dephosphorylation (i.e., activation) of cofilin was with a 3 min incubation with CCK-8, we choose this incubation time in further studies.

### 3.3 Effect of two SSH1 inhibitors, D3 and sennoside A, on CCK/TPA stimulated changes in cofilin and LIMK activation

**FIGURE 3:**
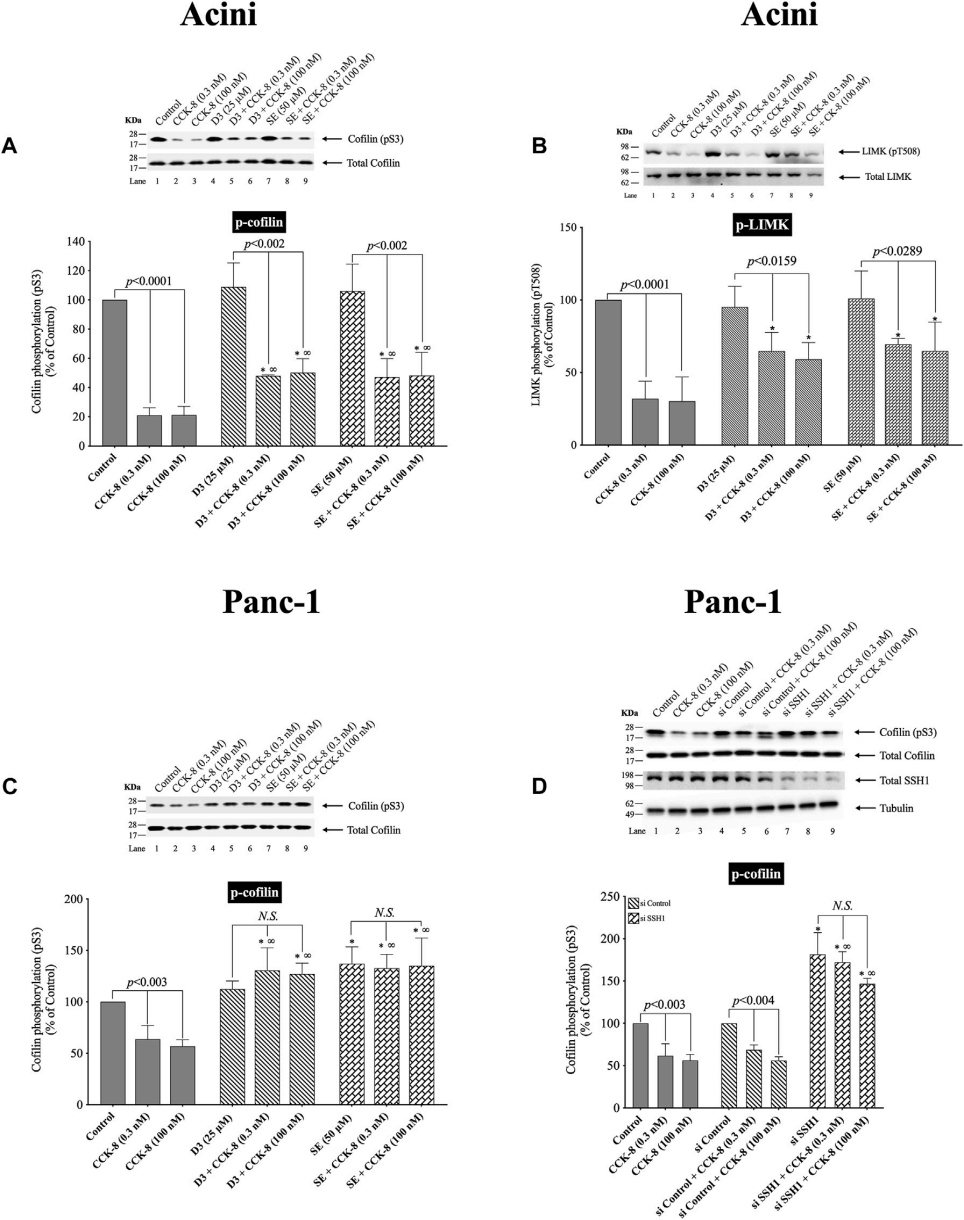
Effect of two SSH1 inhibitors, D3 and SE **(A–C)**, or SSH1 siRNA **(D)**, on the ability of CCK-8 (0.3 and 100 nM) to activate LIMK and cofilin in pancreatic acini **(A/B)** and activate cofilin in CCK_1_-R/Panc-1 cells (**C/D)**. In **(A/B)** isolated pancreatic acini were incubated in the absence or presence of D3 (25 µM) for 1 h or SE (50 µM) for 3 h and then incubated with no addition (control) or CCK-8 (0.3 and 100 nM) for 5 min, and then lysed. In **(C)**, CCK_1_-R/Panc-1 cells were incubated in the absence or presence of D3 (25 µM) for 1 h or SE (50 µM) for 24 h and then incubated with no addition (control) or CCK-8 (0.3 and 100 nM) for 5 min and then lysed. In **(D)**, CCK_1_-R/Panc-1 cells were transfected with siRNA non-targeting Control and siRNA against SSH1 for 48 h, and then incubated with no addition (control) or CCK-8 (0.3 and 100 nM) for 3 min and then lysed. Western blots were analyzed using anti-pS3 cofilin and anti-pT508 LIMK3, and the knockdown effect of SSH1 was confirmed by Western blots using SSH1 (C-term) antibody. Bands were visualized using chemiluminescence and quantified by densitometry. *Top*: Results of a representative blot of four independent experiments are shown. *Bottom*: Means ± S.E. of at least 4 independent experiments. Results are expressed as % of basal stimulation of the control group. *, *p* < 0.05 compared to the control group; ∞, *p* < 0.05 compared to stimulants without inhibitors. *N.S.*, No significant.

(Figure 3) The kinetic results in Figure 2 support the conclusion that neither LIMK nor SSH1 activation is involved in the early activation of cofilin (dephosphorylation), which differs from studies in most tissues with different stimuli, reporting these are the principal signaling cascades involved in cofilin activation (Xu et al., 2021). Unfortunately, it is not possible to do siRNA studies in pancreatic acini, which are frequently used in cultured cell systems, because the acini become de-differentiated and unresponsive during the prolonged incubation times that are required for siRNA effectiveness (Berna et al., 2009). Therefore, we instead used two SSH1 inhibitors; D3 (Li et al., 2015) and SE (Lee et al., 2017), which inhibit the phosphatase activity of the catalytic domain of SSH1 (Li et al., 2015); which are frequently used in studies assessing SSH1’s role in signaling cascades (Lee et al., 2017). In contrast, to a number of nonpancreatic tissues with different stimuli, in which SSH1 activation (dephosphorylation) results in the dephosphorylation (i.e., activation) of cofilin (Spratley et al., 2011; Mizuno, 2013; Doppler et al., 2014), in pancreatic acinar cells, dephosphorylation of cofilin (i.e., activation) caused by CCK-8 (0.3 or 100 nM) was only minimally inhibited by preincubation with either SSH1 inhibitor (SE or D3 by 30%–33%, and 32%–49%, respectively) (Figure 3A, Lane 2–3 vs. five to six and 8–9). Also, preincubation with either SSH1 inhibitor, D3 and SE, only minimally inhibited the CCK-8’s dephosphorylated of LIMK activation by 20% (Figure 3B, Lane 4 vs. 5–6 and 7 vs. 8–9). Neither D3 nor SE affected the basal phosphorylation of LIMK or cofilin (Figures 3A, B, Lane 1 vs. Four and 7). These results support the conclusion from the kinetic phosphorylation experiments involving cofilin, LIMK and SSH1 (Figure 2), that SSH1 activation was not playing a major role in activation of cofilin.

To confirm that the two SSH1 inhibitors (i.e., D3 and SE) at the concentrations used were in fact, effective, we performed a similar study to that on pancreatic acini, investigation the effect of CCK-8 on cofilin and LIMK activation in CCK_1_-R/Panc-1 cells, which allowed a comparative SSH1 siRNA study to also be performed (Figure 3). In CCK_1_-R/Panc-1 cells, similar to pancreatic acini, both concentrations of CCK-8 (0.3 and 100 nM) caused rapidly dephosphorylation of cofilin (i.e., activation) (Figure 3C). In CCK_1_-R -transfected Panc1 cells, both SSH1 inhibitors, D3 and SE, completely inhibited the CCK-8-induced dephosphorylation of cofilin, in contrast to a modest effect in acini (Figures 3C,D). Specifically, SSH1 inhibitors, D3 and SE, completely abolished the dephosphorylation and instead, when CCK-8 (0.3 or 100 nM) was present, a marked elevation in the phosphorylation levels of cofilin occurred (91%–103%) (Figure 3C, Lane 1–3 vs. four to six and 7–9). To confirm our results, we studied the role of SSH1 in CCK-8-induced activation of cofilin by using siRNA against SSH1 in CCK_1_-R/Panc-1 cells (Figure 3D). SSH1 knockdown significantly increased the basal phosphorylation of cofilin by 81% (Figure 3D, Lane 4 vs. 7), and completely inhibited the ability of both CCK-8 concentrations to dephosphorylate cofilin (Figure 3D, Lanes 5, 8, 9), thus showing the siRNA against SSHI had a similar effect to the two SSHI inhibitors. To rule out the possibility that siRNA against SSH1 had a nonspecific effect in the siRNA experiment, we used a non-targeting control-siRNA as a negative control, providing a baseline to compare with the siRNA SSH1 samples (Baum et al., 2010). CCK_1_-R/Panc-1 cells were also incubated with no addition (control) or CCK-8 (0.3 and 100 nM) to study the effect of the non-targeting control-siRNA. These results confirm that the non-targeting control-siRNA had no effect (Figure 3D, Lanes 1–3 vs. 4–6). Unfortunately, we could not study the effect of the SSH1 inhibitors, SE or D3, on LIMK activation in the CCK_1_-R/Panc-1 cells, because T508 LIMK phosphorylation was not detected in these cells (data not shown).

These results further support the conclusion that in pancreatic acinar cells, CCK-8-induced dephosphorylation of cofilin (i.e., activation) and LIMK dephosphorylation (i.e., deactivation) was largely not mediated by alterations in SSH1 activation.

### 3.4 Effect of LIMK inhibitors, LIMKi3 and SR7826, on CCK/TPA stimulated changes in LIMK and cofilin activation

**FIGURE 4:**
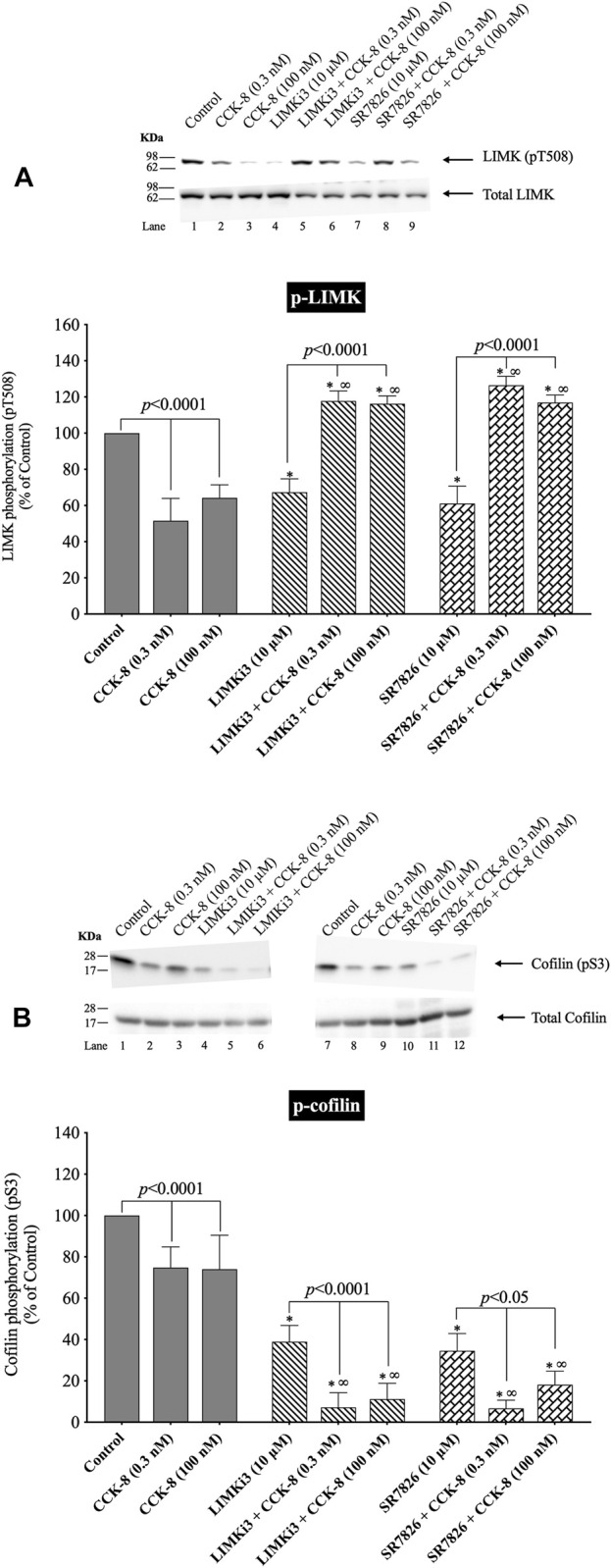
Effect of two LIMK inhibitors, LIMKi3 and SR7826, on the ability of CCK-8 (0.3 and 100 nM) to alter activation of LIMK **(A)** and cofilin **(B)**. Isolated pancreatic acini were incubated in the absence or presence of LIMKi3 (10 µM) or SR7826 (10 µM) for 3 h and then incubated with no addition (control), CCK-8 (0.3 or 100 nM) for 3 min, and then lysed. Western blots were analyzed using anti-pT508 LIMK and anti-pS3 cofilin. Bands were visualized using chemiluminescence and quantified by densitometry. *Top*: Results of a representative blot of four independent experiments are shown. *Bottom*: Means ± S.E. of at least 4 independent experiments. Results are expressed as % of basal stimulation of the control group. *, *p* < 0.05 compared to the control group; ∞, *p* < 0.05 compared to stimulants without inhibitors.

(Figure 4) To further investigate the possible role of alterations in LIMK activation in mediating the rapid dephosphorylation of cofilin (i.e., activation) by CCK-8, we studied the effect of two widely used LIMK inhibitors on CCK-mediated cofilin dephosphorylation (i.e., activation) (Figure 4).

Preincubation with either of two LIMK inhibitors, LIMKi3 and SR7826 (Yu et al., 2018), significantly decreased the basal levels of LIMK by 33% (Figure 4A, Lane 1 vs. 4 and 7), suggesting LIMK activation was an important determinant of basal phosphorylation of LIMK in pancreatic acini. Dephosphorylation of LIMK caused by CCK-8 (0.3 or 100 nM) was completely inhibited by preincubation with both LIMKi3 and SR7826, and in fact, both LIMK inhibitors reversed the pattern of CCK-8’s effect on LIMK phosphorylation, by causing a 128%–145% and 80%, respectively, increase in LIMK phosphorylation with CCK-8 stimulation (Figure 4A, Lane 2 vs. 5–6 and 3 vs. 8–9). Both LIMK inhibitors decreased the basal phosphorylation levels of cofilin by 39% and 67%, respectively (Figure 4B, Lane 1 vs. 4 and 7) suggesting LIMK also regulated basal levels of cofilin, similar to its effect on LIMK. Preincubation with both LIMKi3 and SR7826 did not inhibit the ability of CCK-8 to stimulate dephosphorylation of cofilin (Figure 4B, Lane 2 vs. 5–6 and 3 vs. 8–9).

These results provide additional support that, in contrast to activation of cofilin in numerous other tissues by various stimuli (Xu et al., 2021), in pancreatic acini, LIMK, under our experimental conditions, is not important in mediating CCK-8 induced changes in the phosphorylation levels of cofilin at phosphorylation sites which regulate its activation. However, it is an important determinant of basal levels of phosphorylation of cofilin.

### 3.5 Effect of phosphatases 1A and 2A inhibitors, calyculin a and okadaic acid, and calcineurin inhibitor (PP2B), FK-506, respectively, on CCK-8-stimulated activation of cofilin

**FIGURE 5:**
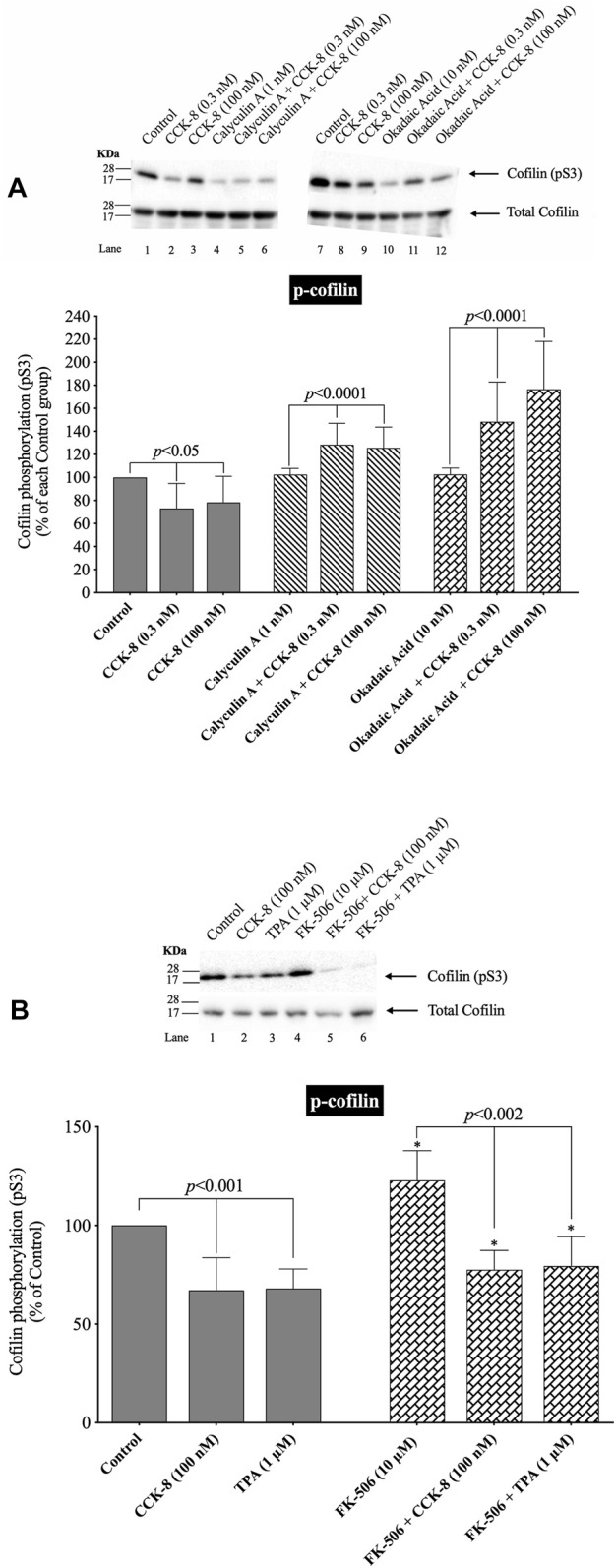
Effect of two serine protein phosphatase inhibitors, calyculin A and okadaic acid, and a Calcineurin inhibitor, FK-5046, on the ability of CCK-8 (0.3 and 100 nM) **(A, B)** or TPA (1 µM) **(B)** to alter the activation of cofilin. Isolated pancreatic acini were incubated in the absence or presence of calyculin A (1 nM), okadaic acid (10 nM) or FK-506 (10 µM) for 3 h and then incubated with no addition (control), CCK-8 (0.3 and 100 nM) for 3 min or TPA (1 µM) for 5 min, and then lysed. Western blots were analyzed using anti-pS3 cofilin. Bands were visualized using chemiluminescence and quantified by densitometry. *Top*: Results of a representative blot of four independent experiments are shown. *Bottom*: Means ± S.E. of at least 4 independent experiments. For **(A)**, results are expressed as % of basal stimulation of each control group. In Figure 4A, calyculin A and okadaic acid decreased the basal level of cofilin phosphorylation by 24% and 12%, respectively. For Figure 4B, results are expressed as % of basal stimulation of the control group with no additions. *, *p* < 0.05 compared to the control group; ∞, *p* < 0.05 compared to stimulants without inhibitors.

**FIGURE 6:**
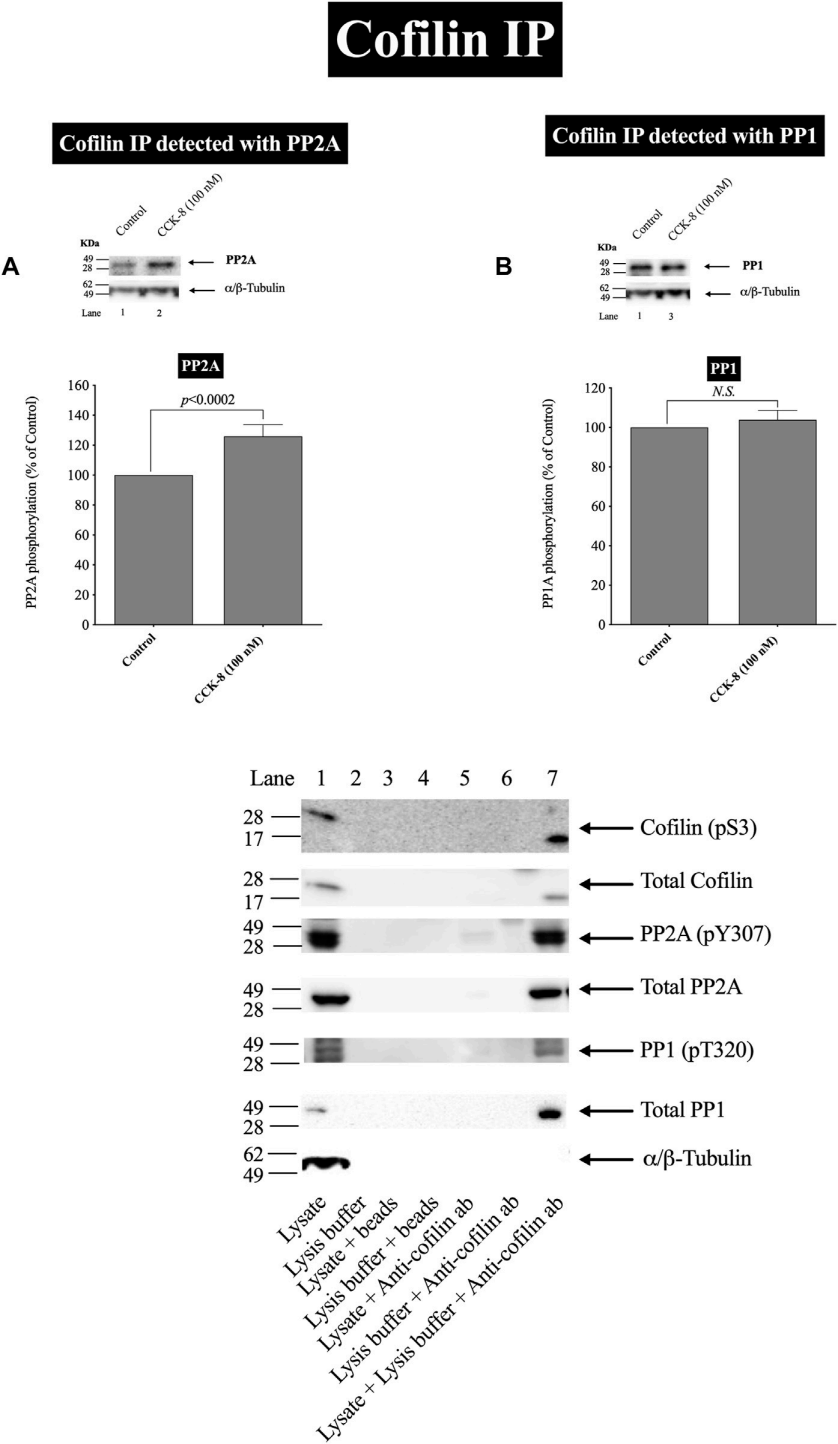
Effect of CCK-8 (100 nM) to alter co-immunoprecipitation of cofilin with total PP2A **(A)** or PP1 **(B)**. Isolated pancreatic acini were incubated in the absence or presence of CCK-8 (100 nM) for 3 min and then lysed. Equal amounts of protein were co-immunoprecipitated with an anti-cofilin (Santa Cruz, SC-376476) and then subjected to Western blots using anti-PP2A and anti-PP1. Bands were visualized using chemiluminescence and quantified by densitometry. *Top*: Results of a representative blot of four independent experiments are shown. *Bottom*: Means ± S.E. of at least 4 independent experiments. Results are expressed as % of basal stimulation of the control group. *N.S.*, No significant. Effect of the lysate, lysis buffer, Protein A/G agarose beads (beads) or Anti-cofilin antibody (Anti-cofilin ab) individually on phospho and total cofilin (pS3), phospho and total PP2A, phospho and total PP1 and tubulin. Lysates were analyzed using cofilin (pS3), Total cofilin, PP2A (pY307), Total PP2A, PP1(pT320), Total PP1 and tubulin. Antibody detecting tubulin was used to verify loading of equal amounts of protein. These results of the experiments shown are representative of 4 others.

(Figures 5, 6) In human T lymphocytes, neurons, the 293 T cell line (human embryonic kidney 293 cells) and HeLa cells, it is reported that activation of cofilin can occur due its dephosphorylation through the co-stimulation activation of accessory receptors (e.g.,.CD2, CD3 or CD28) by CD3xCD28 mAb, with Endothelin-1 (ET-1), and the Ca^2+^ ionophore A23187, which is mediated by activation of serine protein phosphatases (Ambach et al., 2000; Wang et al., 2005; Tam et al., 2019). Therefore, to explores the possible role of the protein phosphatases PP1, PP2A, PP2B in CCK-8-induced activation of cofilin in pancreatic acini, we used a number of specific inhibitors for the protein phosphatases which have been used in other studies for this purpose. Specifically, we used the PP1/PP2A inhibitor, calyculin A, which at low concentrations inhibits equally PP1 and PP2A activity, but not PP2B, and the PP1/PP2A inhibitor, okadaic acid, which a low concentrations has a 50-250-fold greater inhibitory effect on PP2A over PP1 (Ishihara et al., 1989; Takai et al., 1992; Lutz et al., 1993; Schmidt et al., 1994; Walsh et al., 1997), and the PP2B inhibitor, FK-506, which specifically blocks the activation of PP2B (Muili et al., 2013) (Figure 5).

Under our experimental conditions with preincubation with low concentrations of the PP1/PP2A inhibitors, calyculin A and okadaic acid, the activation of cofilin induced by a decrease in phosphorylation level of cofilin stimulated by CCK-8 (0.3 nM and 100 nM), was completely inhibited (Figure 5A, Lane 2–3 vs. five to six and 8–9). In fact, the PP1/PP2A inhibitors reversed the phosphorylation effect of CCK-8, resulting in an increased phosphorylation of cofilin by 53%–71% and 50%–54%, respectively, (Figure 5A, Lane 2–3 vs. five to six and 8–9). In contrast, incubation with the specific PP2B inhibitor, FK-506, had no effect on CCK-8 or TPA’s activation of cofilin (Figure 5B). These inhibitors were both used at low concentrations to take advantage of their different affinities for PP1/PP2A at low concentrations. The fact that both calyculin A and okadaic acid inhibited the CCK-8 induced phosphorylation changes in cofilin at these low concentrations, even though these two inhibitors markedly differ in affinity for PP1, with okadaic acid having low affinity for PP1 and a high affinity for PP2A in other tissues (Ishihara et al., 1989; Ambach et al., 2000), support the likelihood that CCK-8’s activation of PP2A, rather than PP1, was primarily mediating CCK’s effects on cofilin phosphorylation.

In a few other tissues, stimulants activating cofilin through serine/threonine phosphatases (PP1/PP2A) are reported to stimulate initially the association of cofilin with the protein phosphatases (Takuma et al., 1996; Ambach et al., 2000; Oleinik et al., 2010). In order to assess this possibility and confirm further the above results, we studied the ability of CCK-8 (100 nM) to stimulate the possible association of PP2A or PP1 with cofilin. CCK-8 stimulated a 25% increase in PP2A immunoprecipitation with cofilin but had no effect on PP1 co-immunoprecipitation with cofilin (Figures 6A,B). To rule out the possibility that the lysate, lysis buffer, Protein A/G agarose beads or Anti-cofilin antibody had a double cross effect in the co-immunoprecipitation experiment, we study the effect of the lysate, lysis buffer, Protein A/G agarose beads or Anti-cofilin antibody individually on cofilin (pS3), Total cofilin, PP2A (pY307), Total PP2A, PP1(pT320), Total PP1 and tubulin. These results confirm that neither lysate, lysis buffer, Protein A/G agarose beads nor Anti-cofilin antibody, individually, had an effect on co-immunoprecipitation (Figure 6C).

These cofilin co-immunoprecipitation results demonstrate that CCK-8 is increasing the association of PP2A and cofilin, which can lead to alteration in PP2A activation, resulting preferentially in having an increased effect on cofilin activity, as reported in a few other tissues (Takuma et al., 1996; Ambach et al., 2000; Oleinik et al., 2010).

### 3.6 Effect of PKC/PKD and Src, GFX, kbNB and PP2, on CCK/TPA stimulated changes in cofilin activation

**FIGURE 7:**
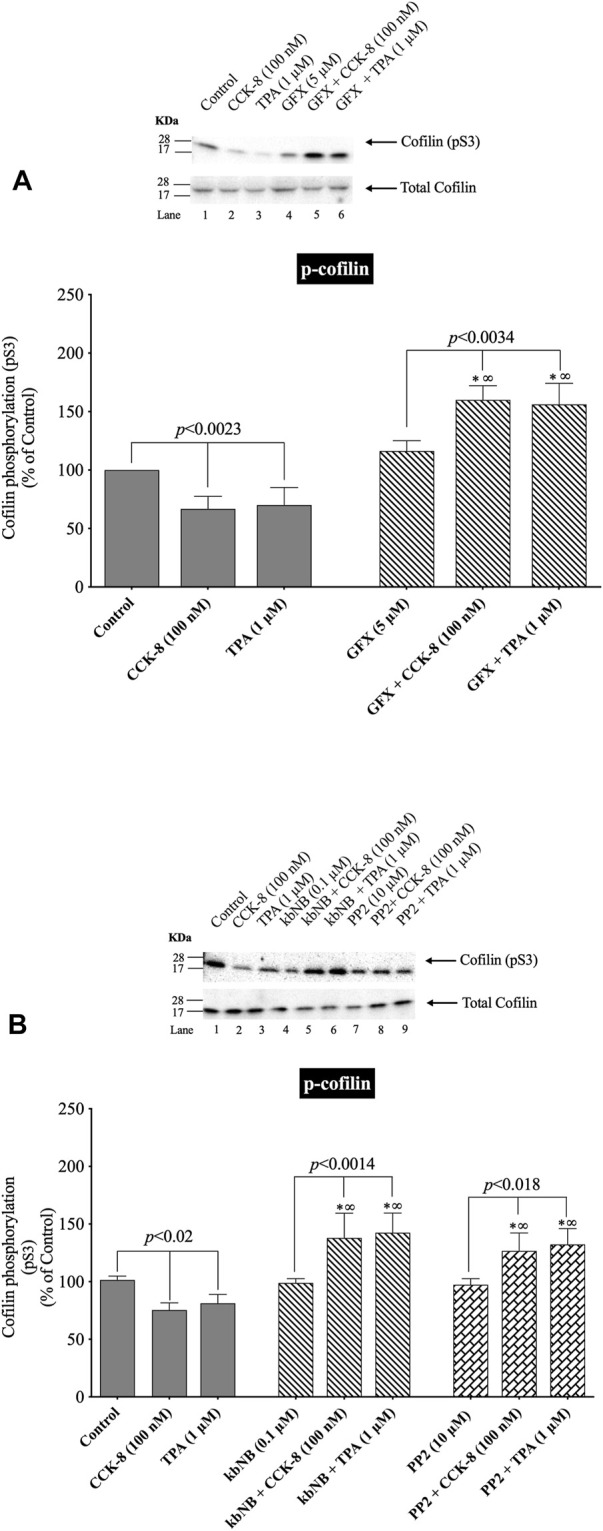
Effect of a PKC inhibitor, GFX; PKD inhibitor, kbNB; or a Src inhibitor, PP2, on the ability of CCK-8 (100 nM) and TPA (1 µM) to alter cofilin activation. Isolated pancreatic acini were incubated in the absence or presence of GFX (5 µM) **(A)**, kbNB (0.1 µM) **(B)** or PP2 (10 µM) **(B)** for 3 h and then incubated with no addition (control), CCK-8 (100 nM, 3 min) or TPA (1 μM, 5 min), and then lysed. Western blots were analyzed using anti-pS3 cofilin. Bands were visualized using chemiluminescence and quantified by densitometry. *Top*: Results of a representative blot of four independent experiments are shown. *Bottom*: Means ± S.E. of at least 4 independent experiments. Results are expressed as % of basal stimulation of the control group. *, *p* < 0.05 compared to the control group; ∞, *p* < 0.05 compared to stimulants without inhibitors.

**FIGURE 8:**
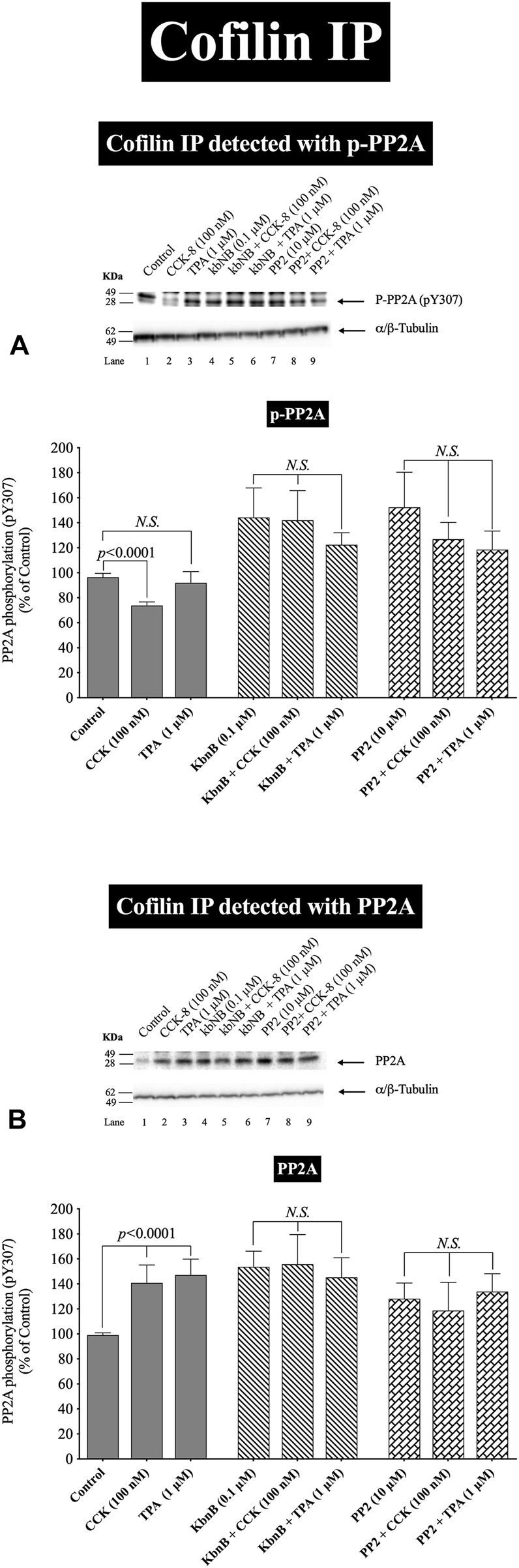
Effect of a PKD inhibitor, kbNB, or a Src inhibitor, PP2, on the ability of CCK-8 (100 nM) and TPA (1 µM) to stimulate the association of cofilin with either phospho-PP2A **(A)** or total PP2A **(B)**. Isolated pancreatic acini were incubated in the absence or presence of kbNB (0.1 µM) or PP2 (10 µM) for 3 h and then incubated with no addition (control), CCK-8 (100 nM, 3 min) or TPA (1 μM, 5 min), and then lysed. Equal amounts of protein were co-immunoprecipitated with an anti-cofilin (Santa Cruz, SC-376476) and then subjected to Western blots using anti-pY307 PP2A and anti-PP2A. Bands were visualized using chemiluminescence and quantified by densitometry. *Top*: Results of a representative blot of four independent experiments are shown. *Bottom*: Means ± S.E. of at least 4 independent experiments. Results are expressed as % of basal stimulation of the control group. *N.S.*, No significant.

(Figures 7, 8) Previous studies in other tissues demonstrated that alteration in PLC/PKC activation can affect the phosphorylation of cofilin resulting in its activation (Zhan et al., 2003; Xu et al., 2015; Wille et al., 2018; Singla et al., 2019). Furthermore, activation of Src kinases and PKD (Eiseler et al., 2009) in other tissues can effect cofilin activation, thus each were investigated for their possible role in mediated cofilin activation with CCK stimulation (Figure 7). This was accomplished by using the PKC inhibitor, GF109203X (GFX); the PKD inhibitor, kbNB142-70, (kbNB), which blocks phosphorylation of PKD and its activation; and the Src inhibitor, PP2, which competes for the ATP binding site on Src and inhibits Src activation. Preincubation with the PKC, PKD or Src inhibitors, GF109203X (Figure 7A), KbNB142-70 (Figure 7B) or PP2 (Figure 7B), respectively, completely reversed cofilin dephosphorylation (i.e., activation) stimulated by CCK-8 and TPA. In addition, the importance of PKC activation in mediating CCK-stimulation of cofilin dephosphorylation (i.e., activation) is also supported by the rapid dephosphorylation induced in cofilin by the PKC activator, TPA (Figures 2B, 5B, 7). None of these inhibitors altered the basal phosphorylation level of cofilin (Figure 7, Lane 1 vs. 4 and 7). These results support the conclusion that activation of both PKC/PKD and Src are each important in mediating CCK-8- and TPA-stimulated changes in cofilin activation.

In order to confirm further the above results, we studied the effect of PKD and Src activation by CCK-8 (100 nM) and TPA to stimulate phospho-PP2A or Total PP2A co-immunoprecipitated with cofilin, which can result in its activation (Zhan et al., 2003; Xu et al., 2015; Wille et al., 2018; Singla et al., 2019). Cofilin co-immunoprecipitation with the inactive phospho-PP2A was decreased with CCK-8 (100 nM), but not with TPA (Figure 8A, Lane 1 vs. 2,3). Preincubation with the PKD or Src inhibitors, GF109203X (Figure 8A) completely inhibited the decrease in phospho-PP2A co-immunoprecipitation stimulated by CCK-8 and TPA (Figure 8A). In contrast, CCK-8 and TPA stimulated a 40% increase in total PP2A co-immunoprecipitation with cofilin (Figure 8B, Lane 1 vs. 2–3), and PKD and Src inhibition reversed this stimulated increase (Figure 8B).

Because PP2A is deactivated by pY307 phosphorylation (Wei et al., 2020), these cofilin co-immunoprecipitation results demonstrate that CCK-8 is altering cofilin activation by regulating its phosphorylation in two ways. First, CCK-8 is increasing the amount of total PP2A associated with cofilin (Figure 8B), which could lead to PP2A activation having an increasing effect on cofilin activity, as shown in Figure 6. Secondly, CCK and TPA are decreasing the fraction of inactive PP2A or conversely, increasing the fraction of the active PP2A, (i.e., nonphospho-PP2A) (Figure 8A) associated with cofilin. Both of these cofilin stimulatory effects of CCK are inhibited by Src and PKD inhibitors. These results support the conclusion that PKD and Src contribute to CCK-8-mediated cofilin activation through PP2A activation.

### 3.7 Effect of PAK4 inhibitors, PF-3758309 and LCH-7749944, to stimulate cofilin

**FIGURE 9:**
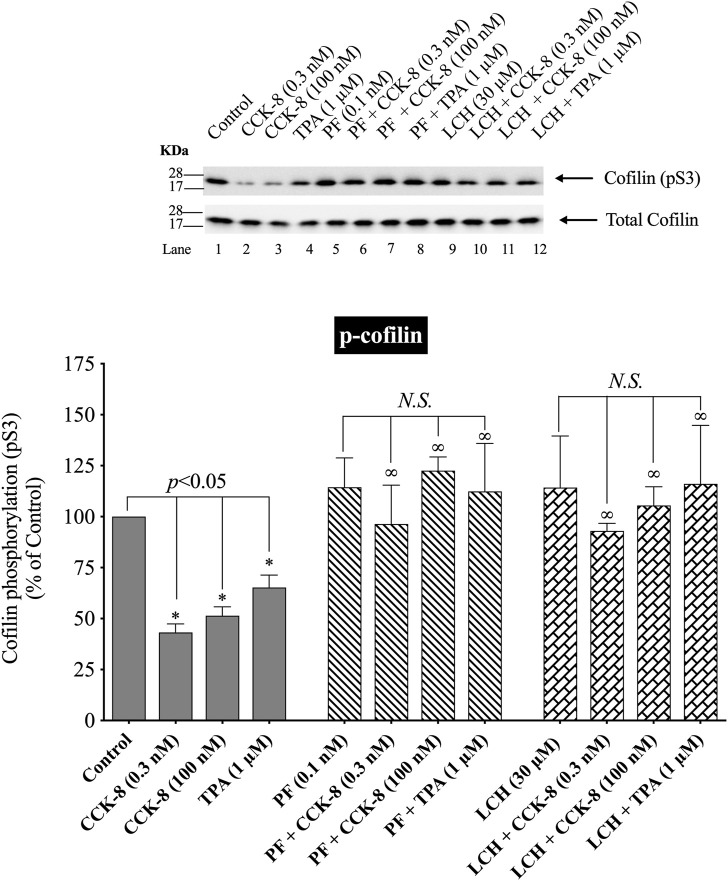
Effect of two PAK4 inhibitors, PF-3758309 or LCH-7749944, on the ability of CCK-8 (0.3 and 100 nM) or TPA (1 µM) to alter activation of cofilin. Isolated pancreatic acini were incubated in the absence or presence of PF-3758309 (0.1 nM) or LCH-7749944 (30 µM) for 3 h and then incubated with no additions (control), CCK-8 (0.3 and 100 nM) for 3 min or TPA (1 µM) for 5 min, and then lysed. Western blots were analyzed using anti-pS3 cofilin. Bands were visualized using chemiluminescence and quantified by densitometry. *Top*: Results of a representative blot of four independent experiments are shown. *Bottom*: Means ± S.E. of at least 4 independent experiments. Results are expressed as % of basal stimulation of the control group. *, *p* < 0.05 compared to the control group; ∞, *p* < 0.05 compared to stimulants without inhibitors; *N.S.*, No significant.

(Figure 9) Numerous previous studies in other tissues with various stimulants, report that activation of p21-activated kinases, include the Group II p21-activated kinase, PAK4, can result in its association with and activation of cofilin (Dan et al., 2001). Furthermore, previous studies report PAK4 kinase is present in pancreatic acinar cells and is activated by CCK-8 (Ramos-Alvarez and Jensen, 2018; Ramos-Alvarez et al., 2019). Therefore, we investigated the role of PAK4 in CCK-8-induced activation of the cofilin in pancreatic acini using two PAK4 inhibitors, PF-3758309 and LCH-7749944, under conditions that have been shown to inhibit specifically PAK4 phosphorylation and activation in these cells (Ramos-Alvarez and Jensen, 2018; Ramos-Alvarez et al., 2019). Both PAK4 inhibitors completely inhibited the dephosphorylation of cofilin stimulated by CCK-8 or TPA (Figure 9, Lane 2–4 vs. 5–8 and 9–12). These results support the conclusion that activation of PAK4 is important in CCK-8-mediated cofilin activation (i.e., dephosphorylation).

### 3.8 Effect of PI3K, p38, JNK and ROCK, on CCK/TPA stimulation of cofilin activation

**FIGURE 10:**
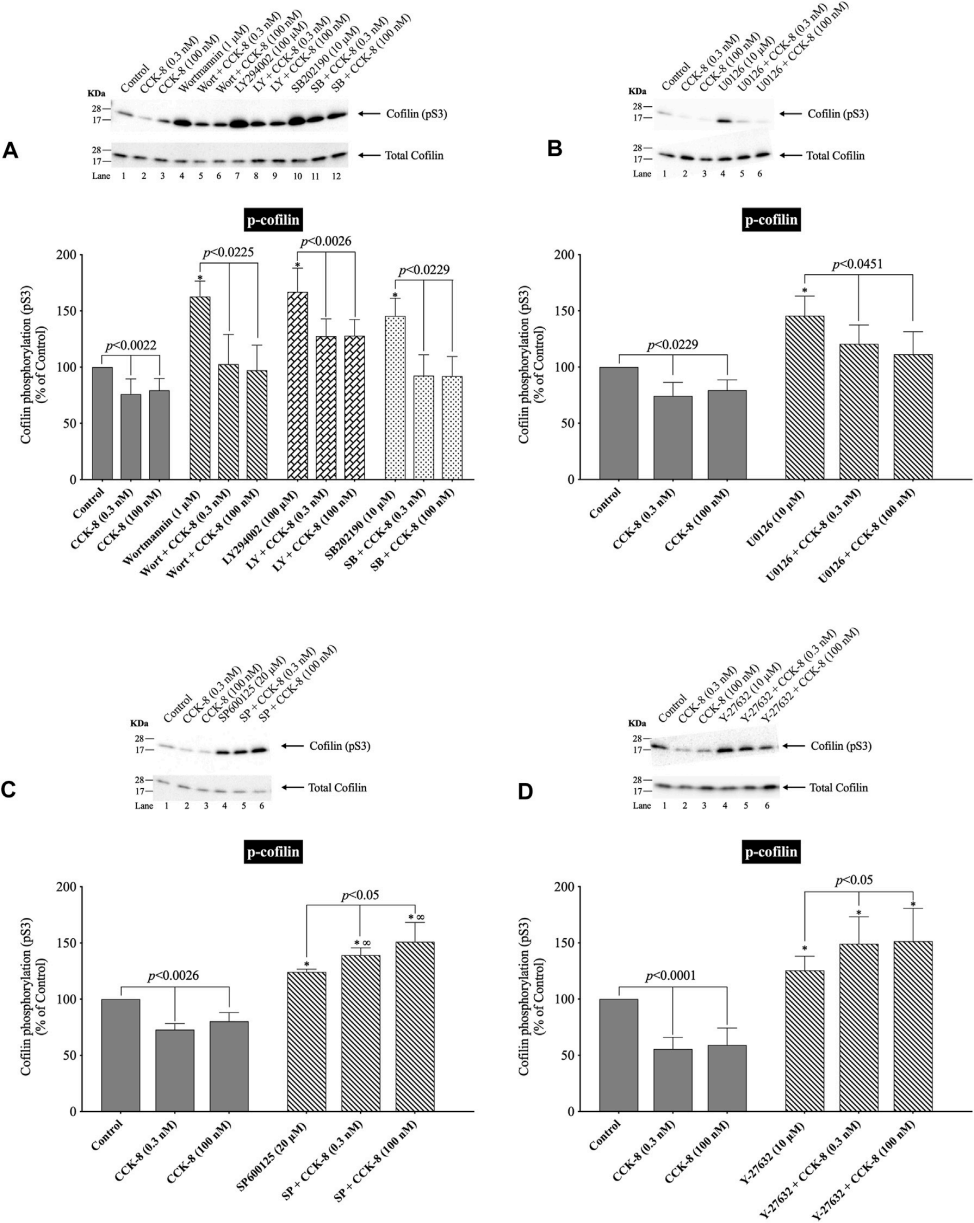
Effect of two PI3K inhibitors, Wortmannin (Wort) and LY294002 (LY); a p38 inhibitor, SB202190 (SB); a MEK inhibitor (U0126); a JNK inhibitor, SP600125 (SP); and a ROCK inhibitor, Y-27632, on the ability of CCK-8 (0.3 and 100 nM) to alter activation of cofilin. Isolated pancreatic acini were incubated in the absence or presence of Wort (1 µM) **(A)**, LY (100 µM) **(A)**, SB (10 µM) **(A)**, U0126 (10 µM) **(B)**, SP600125 (20 µM) **(C)** or Y-27632 (10 µM) **(D)** for 1 h (SP600125, 3 h) and then incubated with no addition (control), CCK-8 (0.3 and 100 nM, 3 min) and then lysed. Western blots were analyzed using anti-pS3 cofilin. Bands were visualized using chemiluminescence and quantified by densitometry. *Top*: Results of a representative blot of four independent experiments are shown. *Bottom*: Means ± S.E. of at least 4 independent experiments. Results are expressed as % of basal stimulation of the control group. *, *p* < 0.05 compared to the control group; ∞, *p* < 0.05 compared to stimulants without inhibitors.

(Figure 10) In various tissues with difference stimuli, cofilin dephosphorylation and activation, has been reported to be mediated by PI3K, ROCK and various MAPKs (p38, p44/42, JNK) (Won et al., 2008; Duan et al., 2014), therefore we assessed their possible role in CCK-8-stimulated cofilin activation. Previous studies have shown that, in pancreatic acini and/or other tissues, CCK-8 activates each of these signaling cascades (Berna et al., 2009; Camello-Almaraz et al., 2009; Ramos-Alvarez and Jensen, 2018; Ramos-Ãlvarez et al., 2020). In order to address the role of these signal cascades on CCK stimulation of cofilin activation, we used, the PI3K inhibitors, Wortmannin and LY294002, which inhibit the ATP-binding site of the catalytic domain of PI3K; the p38 inhibitor, SB202190, which inhibits p38α and p38β with IC_50_ values of 50 and 100 nM, respectively by binding in the ATP binding pocket; the MEK inhibitor, U0126, a non ATP-competitive inhibitor of MEK, which inhibits MEK1 and MEK2 with IC_50_ values of 72 nM and 58 nM, respectively; the JNK inhibitor, SP600125, which is a reversible ATP-competitive inhibitor, which inhibits JNK1-3 with an IC_50_: 0.11 μM; and the ROCK inhibitor, Y-27632, which inhibits both ROCK1 (*Ki*: 220 nM) and ROCK2 (*Ki*: 300 nM) by competing with ATP for binding to its catalytic site (Ishizaki et al., 2000). Only preincubation with the JNK inhibitor, SP600125 (Figure 10C), and the ROCK inhibitor, Y-27632, (Figure 10D), completely reversed cofilin dephosphorylation by CCK-8. Each inhibitor increased the basal phosphorylation of cofilin by 24%–68% (Figures 10A–D). These results support the conclusion that activation of JNK and ROCK, but not PI3K or p38, are important in mediating CCK-8-stimulated changes in cofilin activation.

### 3.9 Effect of the cofilin inhibitors, cytochalasin D and paclitaxel, on CCK/TPA/A71378 stimulation of cofilin activation and activation of p44/42 in pancreatic acini and pancreatic AR42J cells

**FIGURE 11:**
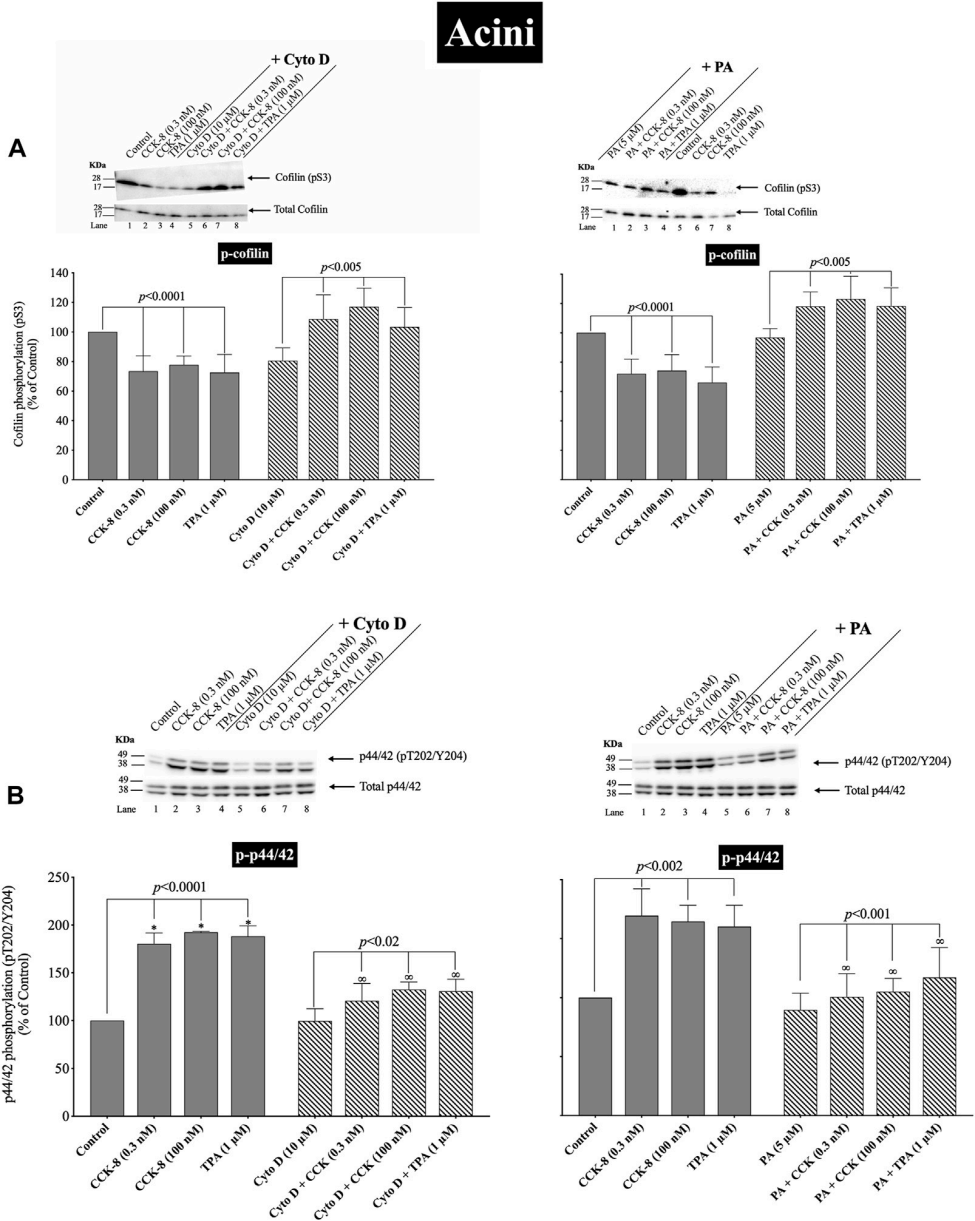
Effect of two cofilin inhibitors, Cytochalasin D or Paclitaxel **(**on the ability of CCK-8 (0.3 and 100 nM) and TPA (1 µM) to alter activation of cofilin **(A)** and p44/42 **(B)**. Isolated pancreatic acini were incubated in the absence or presence of Cytochalasin D (10 µM) or Paclitaxel (5 µM) for 3 h and then incubated with no additions (control), CCK-8 (0.3 and 100 nM) for 3 min or TPA (1 µM) for 5 min, and then lysed. Western blots were analyzed using anti-pS3 cofilin, anti-pT202/Y204 p44/42 and Tubulin. Bands were visualized using chemiluminescence and quantified by densitometry. *Top*: Results of a representative blot of four independent experiments are shown. *Bottom*: Means ± S.E. of at least 4 independent experiments. Results are expressed as % of basal stimulation of the control group. *, *p* < 0.05 compared to the control group; ∞, *p* < 0.05 compared to stimulants without inhibitors.

**FIGURE 12:**
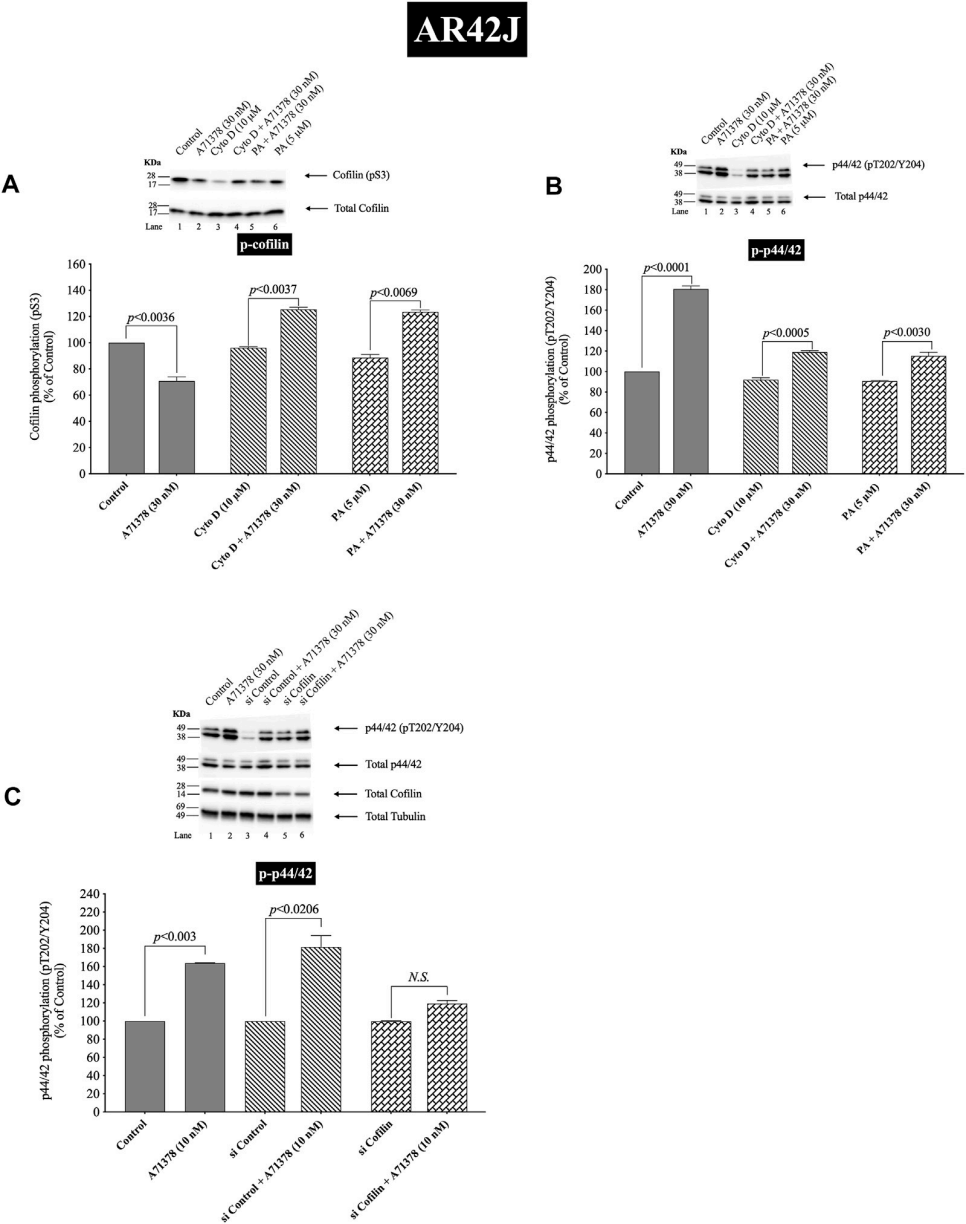
Effect of two cofilin inhibitors, Cytochalasin D or Paclitaxel **(A, B)**, or cofilin siRNA **(C)**, on the ability of A71378 (30 and 10 nM) to alter activation of cofilin **(A)** and p44/42 **(B, C)**. In **(A, B)** AR42J cells were treated with Dexamethasone (100 nM) for 72 h and then incubated in the absence or presence of Cytochalasin D (10 µM) or Paclitaxel (5 µM) for 4 h and then incubated with no additions (control) A71378 (30 nM) for 10 min and then lysed. In **(C)** AR42J cells were treated with Dexamethasone (100 nM) for 72 h and transfected with siRNA non-targeting Control and siRNA against cofilin for 48 h, and then incubated with no additions (control) and A71378 (10 nM) for 10 min and then lysed. Western blots were analyzed using anti-pS3 cofilin, anti-pT202/Y204 p44/42 and Tubulin. Bands were visualized using chemiluminescence and quantified by densitometry. *Top*: Results of a representative blot of four independent experiments are shown. *Bottom*: Means ± S.E. of at least 4 independent experiments. Results are expressed as % of basal stimulation of the control group. *N.S.*, No significant.

(Figures 11, 12) CCK-8 stimulates pancreatic growth in addition to being a physiological regulator of pancreatic enzyme secretion (Jensen, 1994; Dufresne et al., 2006) The activation of p44/42 MAPK by CCK-8 is an important signaling step in mediating its growth effects (Dufresne et al., 2006). Therefore, to study the possible role of cofilin in the activation of p44/42 MAPK in pancreatic acini, we examined the effect on cofilin activation of preincubation with two cofilin inhibitors (Cytochalasin D, Paclitaxel). (Figure 11) (Shoji et al., 2012; Zhang et al., 2018). The concentrations used in this study are: Cytochalasin D at 10 µM and Paclitaxel at 5 µM. These concentrations are similar to studies in fibroblasts in which Cytochalasin D inhibits the interaction between actin and cofilin (Shoji et al., 2012); in epithelial cells where Cytochalasin D completely blocked cell migration (Matsubayashi et al., 2004); and with Paclitaxel which significantly suppressed cofilin-1 expression levels in epithelial cells (Zhang et al., 2018) at micromolar concentrations.

Activation of cofilin induced by pS3 dephosphorylation stimulated by CCK-8 (0.3 or 100 nM) or TPA (1 µM), was completely inhibited by preincubation with either cofilin inhibitor (Cytochalasin D, Paclitaxel) (Figure 11A). Specifically, in contrast to the stimulation of cofilin activation seen without the cofilin inhibitors, preincubation with either cofilin inhibitor (Cytochalasin D, Paclitaxel) resulted in a significant increase in phosphorylation levels of cofilin by CCK-8 (0.3 and 100 nM) or TPA (Figure 11A, Lane 2–4 vs. 6–8), which has been shown in numerous studies (Shoji et al., 2012; Zhang et al., 2018) in other tissues to deactivate cofilin. Furthermore, preincubation with either cofilin inhibitor, (Cytochalasin D, Paclitaxel), significantly decreased the CCK-8- (0.3 and 100 nM) and TPA-mediated activation of p44/42 demonstrated by their ability to inhibit p42/44 phosphorylation levels by 31%–41% and 27%–30%, respectively (Figure 11B, Lane 2–4 vs. 6–8).

To provide support that the above results with both cofilin inhibitors at the concentrations used were in fact not acting nonspecifically at inhibiting p42/44 activation but that it was due to cofilin inhibition, we performed additional studies study using AR42J cells (Figure 12). We studied the effect of A71378, a specific CCK_1_-R agonist, on p44/42 MAPKs activation in AR42J cell by using cofilin inhibitors (Figures 12A,B) and by also using cofilin siRNA to specifically inhibit cofilin expression (Figure 12C). As seen before with CCK-8 in pancreatic acini (Figure 11), activation of cofilin induced by CCK_1_-R was completely inhibited by both cofilin inhibitors, Cytochalasin D, Paclitaxel, (Figure 12A). Preincubation with either cofilin inhibitor, (Cytochalasin D, Paclitaxel), significantly decreased by 34% and 36%, respectively, the A71378 -mediated activation of p44/42 (Figure 12B, Lane 2-*vs.* 4–6). Similarly, specific cofilin knockdown with siRNA inhibited the ability of CCK_1_-R activation stimulate dephosphorylate p44/42 MAPKs (Figure 12C), supporting the specific action of cofilin in mediating CCK stimulated p44/42 MAPK activation. To rule out the possibility that siRNA against cofilin had an effect in the siRNA experiment, we used a non-targeting control-siRNA as a negative control, providing a baseline to compare with the siRNA cofilin samples (Baum et al., 2010). AR42J cells were also incubated with no addition (control) or A71378 (30 nM) to study the effect of the non-targeting control-siRNA. These results confirm that the non-targeting control-siRNA had no effect (Figure 12C, Lanes 1–2 vs. 3–4).

### 3.10 Effect of the cofilin inhibitors, cytochalasin D and paclitaxel, on CCK/TPA/A71378-stimulated amylase release

**FIGURE 13:**
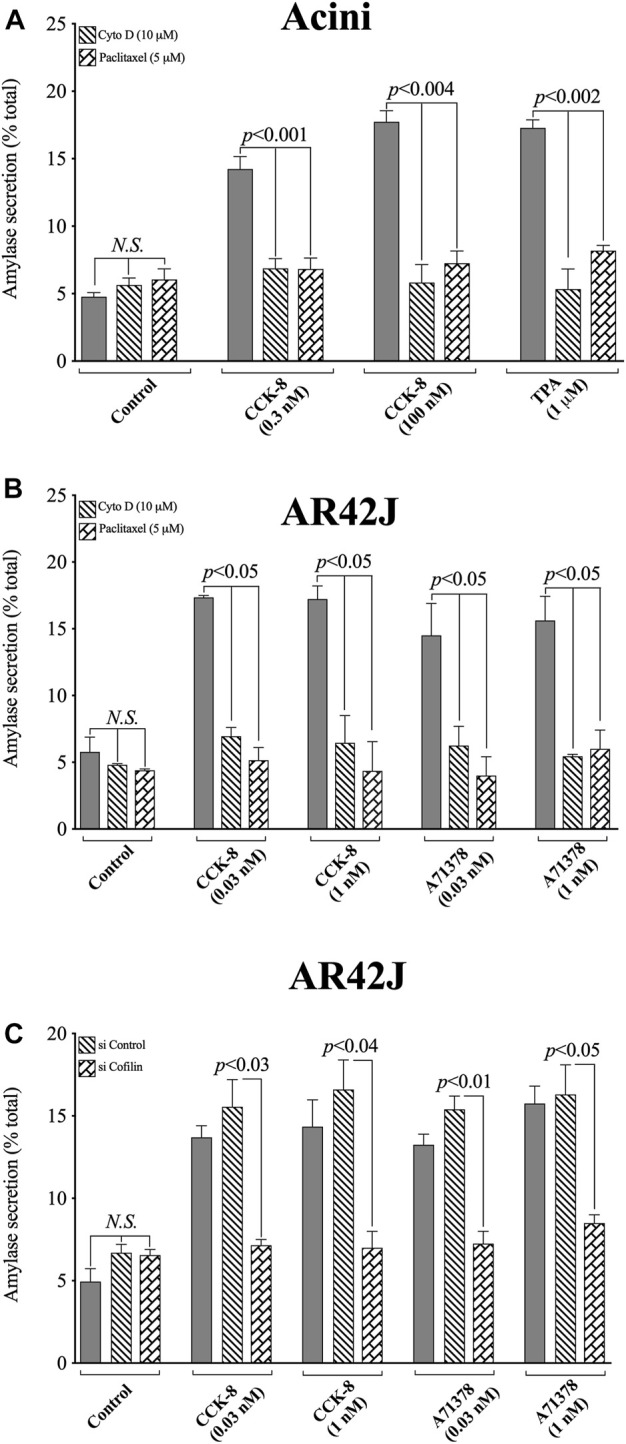
Effect of two cofilin inhibitors, Cytochalasin D or Paclitaxel, **(A, B)**, or Cofilin siRNA **(C)**, on the ability of CCK/TPA/A71378-stimulated amylase release form pancreatic acini **(A)** or AR42J cells **(B, C).** In **(A, B)** pancreatic acini and AR42J cells were incubated in the absence or presence of Cytochalasin D (10 µM) or Paclitaxel (5 µM) for 4 h and then incubated with no additions (control) or CCK-8 (0.03, 0.3, 1 and 100 nM), TPA (1 µM) or A71378 (0.03 and 1 nM) for 30 min and then lysed. In **(B, C)** AR42J cells were treated with Dexamethasone (100 nM) for 72 h, and in **(C)** AR42J cells were transfected with siRNA Non-targeting Control and siRNA against cofilin for 48 h, and then incubated with no addition (control) or CCK-8 (0.03 and 1 nM) and A71378 (0.03 and 1 nM) for 30 min. Amylase release, expressed as percent of cellular total amylase secreted, was determined after 30 min incubation. Means ± S.E. of at least 3 independent experiments. *N.S.*, No significant.

(Figure 13) To assess the possible role of cofilin activation in mediating CCK-stimulated enzyme secretion, we assessed enzyme secretion after incubation with or without CCK-8 (0.03, 0.3, 1 or 100 nM), TPA (1 µM) or A71378 (0.03, 1 nM), the effect of preincubation with either of the two inhibitors of cofilin activation (Figures 13A,B). Both CCK-8 concentrations, as well as TPA alone, resulted in a stimulation of pancreatic acinar enzyme secretion, as previously reported (Ramos-Alvarez and Jensen, 2018), with CCK-8 caused a 207%–287% (0.3 and 100 nM, respectively) and TPA a 278% increase, in amylase release (Figure 13A). Pretreatment with either of the cofilin inhibitors (Cytochalasin D, Paclitaxel) completely inhibited the pancreatic acinar amylase secretion stimulated by both concentrations of CCK-8, and of TPA (Figure 13A).

To confirm that the two cofilin inhibitors (i.e., Cytochalasin D and Paclitaxel) at the concentrations used were in fact, having a specific inhibitory effect on cofilin, we performed a similar study to that on pancreatic acini, investigation the effect of CCK-8 and the CCK_1_-R specific agonist, A71378 on amylase release in AR42J cells with the two cofilin inhibitors (Figures 13A,B), as well as performing a comparative cofilin siRNA study (Figure 13C).

Similar to pancreatic acini, in AR42J cells, both cofilin inhibitors, Cytochalasin D and Paclitaxel, completely inhibited the CCK-8/A71378-induced amylase secretion, (Figure 13B). Similarly, specific cofilin knockdown using cofilin siRNA, completely inhibited the ability of both CCK-8 and A71378 at both concentrations to stimulate amylase secretion (Figure 13C). Neither of these two cofilin inhibitors influenced basal amylase release (Figures 13A,B). To rule out the possibility that siRNA against cofilin had an effect in the siRNA experiment, we used a non-targeting control-siRNA as a negative control, providing a baseline to compare with the siRNA cofilin samples (Baum et al., 2010). AR42J cells were also incubated with no addition (control) or CCK-8 (0.03 and 1 nM) and A71378 (0.03 and 1 nM) to study the effect of the non-targeting control-siRNA. These results confirm that the non-targeting control-siRNA had no effect (Figure 13C). These results support the conclusion that amylase secretion stimulated by CCK-8, TPA or A71378 requires activation of cofilin.

## 4 Discussion

In the present study, to explore a possible role for cofilin in mediating CCK’s effects on acinar cell function, we first investigate CCK’s ability to activate cofilin followed by investigating the possible signaling cascades involved in the activation and finally we investigated its effects on CCK mediated enzyme secretion and in stimulation of the p42/44 cascade essential for CCKs growth effects in both pancreatic acini and pancreatic AR42J cells. We performed the initial studies by using different chemical inhibitors for LIMK (Yu et al., 2018), cofilin (Shoji et al., 2012; Zhang et al., 2018), SSH1(Li et al., 2015; Lee et al., 2017), and as well as inhibitors of the phosphatases PP1A and PP2A (Takai et al., 1992; Schmidt et al., 1994), which are known to alter cofilin activation in other tissues with other stimuli (Spratley et al., 2011; Doppler et al., 2014; Ohashi, 2015; Xu et al., 2021). We also used inhibitors of signaling cascades that CCK is known to activate, and which have been reported to alter cofilin activity in other systems by other stimuli. These different inhibitors were used to investigate the signaling cascades possible involved, because functional, dispersed pancreatic acini cannot be maintained for more than 1 day in culture and retain their full functionality, precluding the use of siRNA inhibition studies in the acini. Similarly, to confirm the results of the effects of cofilin inhibitors on CCK induced enzyme secretion or stimulation of the p42/44 growth cascade in pancreatic acini, we repeated the experiments using AR42J cells which allowed specific cofilin siRNA inhibition studies, as well as cofilin inhibitor studies.

Our initial study showed that secretagogues stimulating PLC-activated cascades resulting in changes of cytosolic calcium and PKC activation (CCK, carbachol, and bombesin) (Jensen and Gardner, 1981; Jensen, 1994; Williams, 2019); and those activating adenylate cyclase, resulting in increases in cellular cAMP (secretin and VIP) (Yan et al., 2016) stimulated activation in pancreatic acini. These results are similar to results in other tissues which report stimulants activating PLC-cascades as well as those activating cyclic AMP cascades can stimulate cofilin activation (Zhan et al., 2003; Karlsson et al., 2010; Xu et al., 2015; Wille et al., 2018) and our results with carbachol in pancreatic acini are also similar to its ability to activate cofilin in rat parotid cells (Takuma et al., 1996).

Previous studies have shown that the CCK_1_ receptor can exist in both a high- and low-affinity state, which can mediate different cellular responses (Sato et al., 1989; Stark et al., 1989; Tapia et al., 1999; Berna et al., 2007). Our results demonstrate that activation of both receptor sites is required for maximal cofilin activation. This conclusion is supported by the results with CCK-8-JMV, which is a full agonist for the high-affinity CCK_1_ receptor state and an antagonist for the low-affinity state in rat pancreatic acini (Sato et al., 1989; Stark et al., 1989). These results are similar to CCK-stimulated of different pathways such as PAK4 (Ramos-Alvarez and Jensen, 2018) and PAK2 activation (Nuche-Berenguer and Jensen, 2015), Src kinases (Lyn and Yes) (Pace et al., 2006), the focal adhesion kinases (p125FAK and PYK2) (Tapia et al., 1999), in pancreatic acini, which require activation of high and low CCK_1_-R affinity sites for full activity. However, our results differ from results with CCK-stimulated activation of PLC or PI3K, which require only high-affinity CCK_1_ receptor state activation (Rivard et al., 1994).

To explore the cellular signaling cascades involved in cofilin activation our initial studies examining the kinetics of the phosphorylation of the activation sites of LIMK, cofilin and SSH1 stimulated by CCK_1_-R activation, because in many other tissues with different stimuli these are the main signaling molecules regulating cofilin activity through its phosphorylation (Ohashi, 2015; Xu et al., 2021). These studies demonstrated an initial activation of cofilin, followed by a loss of activation over time, which is similar to the effect on cofilin activation by angiotensin II in HeLa cells (Kim et al., 2009); PDGF in human aortic smooth muscle cells (Won et al., 2008); and FMLP in peripheral blood leukocytes (Okada et al., 1996). However, this pattern of activation followed by deactivation of cofilin by CCK differs from the effect of LH stimulation in granulosa cells (Karlsson et al., 2010) or NGF or insulin in HT4 neurons (Meberg et al., 1998), where the cofilin activation (dephosphorylation) is maintained for prolonged times. Furthermore, our results showed that, in contrast to cofilin and LIMK, CCK only stimulated increased inactivation of SSH1 (Xu et al., 2021). These results demonstrate that both CCK and TPA can alter the activation of cofilin, LIMK and SSH1 and its effect varies with time.

A number of our results support the conclusion that CCK-mediated activation of cofilin in pancreatic acini is not being mediated by the principal signaling cascades generally regulating cofilin phosphorylation state (i.e., LIMK/SSH1) in a number of other tissues with other stimulants (Spratley et al., 2011; Doppler et al., 2014). First, the kinetic study demonstrated CCK only deactivation of SSH1 (Xu et al., 2021), when maximal cofilin activation occurred (i.e., <1–2 min). Second, although CCK stimulated a de-activation of LIMK, this alone without a commitment activation of a phosphatase, could not account for cofilin’s rapid dephosphorylation. Third, the two SSH1 inhibitors had minimal effect on the CCK-induced cofilin activation as well as the deactivation of LIMK, which in other cell systems with other stimulants can be also affect SSH1 activation (Spratley et al., 2011; Doppler et al., 2014). Fourth, neither of the two LIMK inhibitors altered CCK-induced activation of cofilin. However, each of these LIMK inhibitors reversed CCK-induced alterations of LIMK activation, demonstrating their effectiveness and providing additional support for the conclusion that alterations in LIMK activity by CCK were not contributing to the activation of cofilin. These results with CCK are in contrast to results in a number of other tissues where stimulants activating cofilin did so through a SSH1 activation mechanism, such as with PDGF stimulation of in human aortic smooth muscle cells (Won et al., 2008); with hepatocyte growth factor in macrophages (Singla et al., 2019) or FMLP activation of cofilin in leukocytes (Okada et al., 1996). These results support the conclusion that CCK in pancreatic acini is stimulating cofilin activation by activating another phosphatase different from SSH1, and thus differs from the effects of numerous stimulants in a number of other cells wherein the dephosphorylation and activation of cofilin is primarily due to activation of SSH1 (Spratley et al., 2011; Mizuno, 2013; Doppler et al., 2014).

Because our results differ from the general finding that the two major signaling cascades in other tissues mediating cofilin activity, which involve alterations in LIMK and/or SSH1 activity (Wang et al., 2005; Spratley et al., 2011; Mizuno, 2013; Ohashi, 2015; Xu et al., 2021), were not important in mediating CCK’s activation of cofilin in pancreatic acini, we explored the possible involvement of other phosphatases that have been reported to participate in cofilin activation in other tissues with other stimulants. The activation of the serine/threonine phosphatases type 1 (PP1) or type 2A (PP2A) is reported to play an important role in mediating cofilin activation in HeLa cells with the Ca^2+^ ionophore A23187 (Wang et al., 2005) and primary hippocampal neurons with endothelin-1 (ET-1) (Tam et al., 2019). Furthermore, the activation of calcineurin (PP2B) is reported to be involved in the dephosphorylation of cofilin by a number of stimulants in other cells by various stimulants (Meberg et al., 1998; Pandey et al., 2009; Tam et al., 2019). A number of our results support the conclusion that activation of a serine/threonine phosphatase (PP1, PP2A) but not calcineurin, mediates CCK-8-stimulated dephosphorylation of cofilin and its activation in pancreatic acini. This conclusion is supported by the finding that the PP1/PP2A inhibitors, calyculin A and okadaic acid (Ishihara et al., 1989; Takai et al., 1992; Lutz et al., 1993; Schmidt et al., 1994; Walsh et al., 1997), completely inhibited CCK-8-mediated dephosphorylation of cofilin in pancreatic acini. In contrast, the PP2B/calcineurin inhibitor, FK-506 (Muili et al., 2013), had no effect. Furthermore, nanomolar concentrations of okadaic acid, which has been shown in pancreatic acini (Lutz et al., 1993; Bi et al., 2005) and other tissues (Ishihara et al., 1989; Walsh et al., 1997) to be selective for PP2A over PP1, as well as the non-selective PP1/PP2A inhibitor calyculin (Ishihara et al., 1989), each inhibited CCK-8-induced dephosphorylation of cofilin, suggesting that PP2A was primarily responsible for modulating CCK-8-induced cofilin dephosphorylation (i.e., activation). This result is consistent with the finding in pancreatic acinar cells that the predominant CCK_1_R-stimulated phosphatase activity found in the cytosol is PP2A (Lutz et al., 1993) and this is potently inhibited by okadaic acid (IC_50_ = 0.2 nM) (Lutz et al., 1993). Furthermore, in pancreatic acini at nanomolar concentrations, okadaic acid was found to only inhibit PP2A (Wagner et al., 1992). This conclusion is further supported by the finding that CCK stimulated the association of PP2A, not PP1, with cofilin. This association has been reported in a number of other tissues with various stimulants to occur prior to the dephosphorylation and activation of cofilin (Takuma et al., 1996; Ambach et al., 2000; Oleinik et al., 2010). Our results are consistent with a number of studies in various cells which report activation of PP1/PP2A can mediated activation of cofilin (Ambach et al., 2000). Furthermore, studies in human leukemia cells, parotoid acinar cells, and human T lymphocytes (Takuma et al., 1996; Ambach et al., 2000) report that PP1/PP2A can interact directly with cofilin, this interaction is stimulated by agents activating cofilin, and that the activation of the cofilin can be inhibited by PP1/PP2 inhibitors (Ambach et al., 2000). Even though calcineurin (PP2B) is a member of the serine/threonine phosphatase family, under our experimental conditions, cofilin activation is independent from calcineurin activation. Similar to our results the calcineurin inhibitor, FK-506, did not affect the dephosphorylation of cofilin in human T lymphocytes stimulated by CD2 or CD28 (Ambach et al., 2000). However, in contrast to our results, calcineurin dephosphorylated SSH1 and increased the cofilin-phosphatase activity of SSH1 in 293 T and HeLa cells (Wang et al., 2005).

In addition to LIMK/SSH1, a number of other signaling cascades have been described as an important upstream regulator of cofilin activation in various tissues with different stimuli (Won et al., 2008; Duan et al., 2014), including PKC/PKD, Src (Zhan et al., 2003; Xu et al., 2015; Wille et al., 2018; Singla et al., 2019), PAK4 (Dan et al., 2001; Pandey et al., 2009; Mizuno, 2013) and the MAP kinase family. Although CCK is known to activate each of these different signaling cascades (i.e., PKC/PKD, Src, PAK4, MAPKs (Jensen and Gardner, 1981; Ramos-Alvarez and Jensen, 2018) and their activation in pancreatic acini is important in mediating various CCK stimulated functions (i.e., growth, secretion, etc.) (Jensen and Gardner, 1981; Ramos-Alvarez and Jensen, 2018; Ramos-Alvarez et al., 2019; Ramos-Ãlvarez et al., 2020), there are no studies in pancreatic acinar on their possible role in CCK-mediated cofilin activation. Therefore, each of the signaling cascades was investigated for the effect of their activation by CCK on cofilin activation (i.e., dephosphorylation).

Our results support the conclusion that CCK-8 and TPA stimulation of PKC, PKD, Src, PAK4, JNK and ROCK, but not MEK, p38 or PI3K, are required for cofilin activation in pancreatic acinar cells. These results have similarities and differences from the signal cascades reported to mediate cofilin activation by other stimuli in other tissues. Our results demonstrating PLC/PKC activation are important in CCK stimulation of cofilin are similar to that reported in numerous other tissues with other stimuli (Xu et al., 2015; Wille et al., 2018; Singla et al., 2019). However, our results also different from most of these tissues, in which cofilin activation is generally reported by PLC/PKC to be mediated by its activation of SSH1, such as in macrophages with HGF (Singla et al., 2019) or in neutrophils with fMLP (Xu et al., 2015; Wille et al., 2018). In contrast, activation of PLC/PKC did not activate SSH1 in our studies, but instead activated protein phosphatases (PP1 and PP2A, likely PP2A) to activate cofilin. In this respect, our results are similar to that seen with cofilin activation in skeletal muscle cells with insulin (Srinivasan and Begum, 1994) or neutrophils with fMLP (Djafarzadeh and Niggli, 1997). Our results with CCK stimulation of Src being required for CCK activation of cofilin are similar to stimulation of cofilin in macrophages with opsonized zymosan (Matsui et al., 2001) and in aortic smooth muscle stimulated by PDGF (Won et al., 2008). However, they differ from results in osteoblasts stimulated by adhesion (Zambuzzi et al., 2009) or with integrin α5 activation of epithelial cells (Oh et al., 2007) in which Src-mediated inactivation of cofilin.

Our results demonstrating that PAK4 and PKD are required for CCK-stimulated activation of cofilin differ from results in most other cells with other stimuli (Eiseler et al., 2009; Olayioye et al., 2013). In numerous tissues with various stimuli, PKD has been shown to activate PAK4, which in term leads to activation of LIMK resulting in cofilin deactivation (Dan et al., 2001; Spratley et al., 2011; Mizuno, 2013; Olayioye et al., 2013). In addition, PAK4 inactivates SSH1, which also deactivates cofilin (Eiseler et al., 2009; Spratley et al., 2011; Olayioye et al., 2013). However, our results with PAK4 stimulating activation of cofilin are similar to the effects of insulin in skeletal muscle (Tunduguru et al., 2014) and thrombin activation of platelets (Pandey et al., 2009). Our results with PKD activation are similar to results with PKD activation in Hela cells and in fibroblasts (Doppler et al., 2014). Our results demonstrate that CCK activation of JNK kinase, but not ERK or p38, is also required for cofilin activation. Activation of each member of the MAPKs (i.e., JNK, p38, ERK) has been shown to effect cofilin activation in various other tissues with different stimuli (Won et al., 2008; Spratley et al., 2011). Our results with JNK required for cofilin activation are similar to the effect of PGDF in aortic smooth muscle cells (Won et al., 2008), cerulenin in leukemia cells (Zhang et al., 2016) and for neuronal axon elongation during development (Sun et al., 2013). However, they differ from effects of angulin-1 on cofilin activation in endometrial cancer cell (Konno et al., 2020), in which JNK activation caused cofilin deactivation. These results also differ from the role of p38 activation in stimulating growth of breast cancer by inactivating cofilin (Xu et al., 2012). Our results demonstrating that CCK-mediated cofilin activation required ROCK are similar to studies in dorsal root ganglia where *δ* opioids stimulate cofilin activation by ROCK activation (Mittal et al., 2013); in fibroblasts, where ROCK activation is required for stabilizing actin cytoskeleton through regulating cofilin phosphorylation (Shi et al., 2013). However, our results differ from studies in breast cancer cells (Peng et al., 2019) or the effects of hyperosmotic stress in keratinocytes (Silva et al., 2015), in which ROCK increased phospho-cofilin (i.e., inactivation), or studies in which ouabain inhibits Na^+^K^+^-ATPase activity by decreasing ROCK activation, which resulted in cofilin activation (Jung et al., 2006).

In our study CCK stimulated activation of the PI3K cascade was not required for cofilin activation, which differs from its important role in most tissues for PI3K activation in mediating cofilin stimulation (Mizuno, 2013). This includes insulin activation of 293 cells and other cells (Mizuno, 2013) and PDGF activation of NIH 3T3 cells (Nebl et al., 2004). In contrast, our results of lack of effect of PI3K signaling on cofilin activation are uncommon, with only two other studies with similar results (Pandey et al., 2009; Vitolo et al., 2013). The above results demonstrate that the signaling cascades mediating CCK activation of cofilin in pancreatic acinar cells show a number of similarities as well as differences, from what is frequently reported in other tissues with other stimuli.

Previous studies show that activation of cofilin has a key role in mediating secretion in different tissues (Mizuno, 2013), including insulin secretion (Jayaram and Kowluru, 2012), parotoid exocrine secretion (Takuma et al., 1996), platelet degranulation (Pandey et al., 2009) and histamine release from basophilic leukemia cells (Sakuma et al., 2012). Even though there is no information on the role of cofilin activation and secretion in pancreatic acinar cells, other studies provide evidence for an important role for the actin cytoskeleton in modulating secretory granule exocytosis in pancreatic and rat parotoid acinar cells (Messenger et al., 2014); as well as the Rho family small G proteins RhoA and Rac1 regulating secretion through remodeling of the actin cytoskeleton in the pancreas (Williams et al., 2009). Because in other cells with numerous other stimuli, cofilin functions as an essential actin regulatory protein for modulating actin’s activation (Ohashi, 2015; Xu et al., 2021), one would predict cofilin activation could play a major role in pancreatic secretion, and possibly growth, as we have found in this study.

Our results, by using two cofilin inhibitors, Cytochalasin D and Paclitaxel (Shoji et al., 2012; Zhang et al., 2018), show that cofilin activation is required for amylase secretion in pancreatic acinar cells. This result was further verified in pancreatic acinar AR42J cells in which both of these cofilin inhibitors, as well as specific cofilin siRNA studies, further demonstrated the importance of cofilin activation for pancreatic enzyme secretion. These results and our findings that activation of PP2A can mediate activation of cofilin, when combined with results from older studies, support an important role for cofilin activation in CCK stimulated secretion in pancreatic acini. Specifically, in older studies activation of serine/threonine phosphatases (PP1/PP2A) was shown to be required for stimulation of amylase by CCK-8 in pancreatic acinar cells (Schmidt et al., 1994). Furthermore, okadaic acid, which at low concentrations has a 50-250-fold greater inhibitory effect on PP2A over PP1 (Ishihara et al., 1989; Takai et al., 1992; Lutz et al., 1993; Schmidt et al., 1994; Walsh et al., 1997), completely blocked stimulated enzyme secretion in pancreas (Waschulewski et al., 1996) and disrupted amylase release in parotid acinar cells (Tamaki and Yamashina, 2002). Each of these results are compatible with our results, suggesting a distal effect of CCK-8-mediated cofilin activation in secretion through PP2A.

A number of studies in other tissues with other stimuli demonstrate that cofilin is involved frequently in growth/proliferation in both normal and neoplastic tissues (Werle et al., 2021). Studies suggest cofilin is also important in pancreatic cancer growth (Werle et al., 2021), however there are no studies of its involvement in growth/proliferation of normal pancreatic acinar tissue. Our results, by using the two cofilin inhibitors, Cytochalasin D and Paclitaxel (Shoji et al., 2012; Zhang et al., 2018), in pancreatic acini, as well as in the pancreatic acinar cell line, AR42J, with cofilin siRNA treatment to knockdown cofilin levels, show that the ability of CCK_1_-R activation to stimulate p42/44 MAPK activation, a key step in mediating pancreatic acinar growth/proliferation (Dufresne et al., 2006), is dependent on the activation of cofilin. The above results combined with our finding that in pancreatic acini the CCK- mediated activation of cofilin is critically dependent on activation of the protein phosphatase, PP2A, is consistent with results of some previous studies. A previous study (Sans et al., 2004) demonstrated in pancreatic acini the ability of CCK_1_-R activation to stimulate translation elongation, a key step in mediating protein synthesis/growth, is regulated by eEF2 through the mTOR, p38, and MEK pathways, and modulated through PP2A, which can activate cofilin in these cells. These results, support the conclusion that cofilin activation is important for meditating CCK induced protein synthesis/growth/proliferation.

Our study has one potential major weakness in the exploration of the role of LIMK/SSH1 in cofilin activation and the role of cofilin in pancreatic acinar secretion or growth. Because we could not use siRNA in dispersed pancreatic acini, with the prolonged incubation required resulting in unresponsive cells, we had to rely on inhibitors, which even though widely used in the literature, had limited selectivity and could cause off target effects. We dealt with this by using both the inhibitors and siRNA in AR42J cells or CCK1-R/Panc-1 cells and showed identical responses to what we obtained in acini with the inhibitors alone. While this cannot completely rule out the possibility of off target effects of the inhibitors, with the identical results with siRNA studies and the inhibitors in these cells, this makes that conclusion less of a possibility. In the future to confirm our results a different approach could be used such as using acinar cells from the pancreas-specific cofilin KO mice.

**FIGURE 14:**
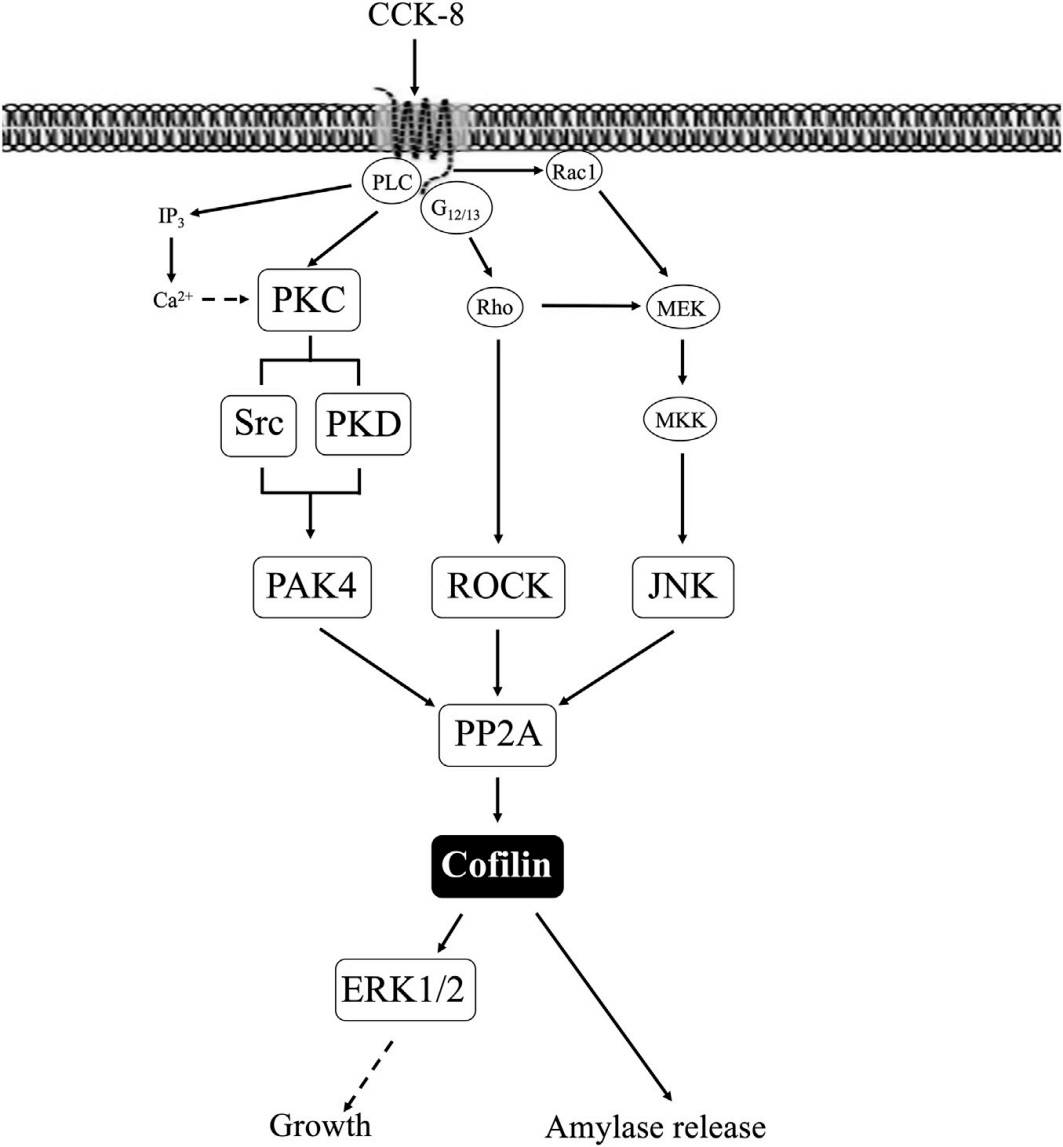
Schematic diagram of signaling cascade for activation of cofilin in pancreatic acinar cells. In rat pancreatic acinar cells, maximal activation of cofilin by cholecystokinin (CCK)-8 requires activation of PKC, which mediates both Src family of kinase (SFK) and protein kinase D (PKD) activation resulting in PAK4 activation; requires activation of ROCK, which is mediated by Rho; requires activation of JNK, mediated by activation of this MAPK pathway; and requires activation of serine protein phosphatases (PP2A), which are a substrate of PKC. Cofilin activation is important for CCK-stimulated enzyme secretion as well as ERK1/2 activation which has been shown to mediate growth. Squares represent signaling pathways shown to be involved in cofilin activation in this study.

In conclusion, the results of the present study demonstrating CCK-mediating activation of cofilin and the signaling cascades involved, can be as summarized as shown diagrammatically as in Figure 14. Our initial studies show that usual mediators of cofilin activation seen in other tissues with other stimuli, involving LIMK and SSH1, are not mediating the effect of CCK on cofilin activation in pancreatic acini. However, we demonstrate that cofilin activation (i.e., dephosphorylation) is mediated by activation of PKC/PKD, Src, PAK4, JNK, ROCK and activation of protein serine/threonine phosphatases (PP1/PP2A, likely PP2A). Numerous previous studies have established one of the principal signaling cascades mediating the cellular effects of CCK_1_R activation on pancreatic acinar cells is the activation of PLC, which results in the mobilization of cellular calcium, and PKC activation (Tapia et al., 2002; Berna et al., 2007), which in turn activate Src kinases and PAK4 (Ramos-Alvarez and Jensen, 2018). Furthermore, CCK stimulation in these cells can result in activate of all MAPKs (ERK, JNK, p38) (Williams, 2001). However, activation of p38 and MEK were not important in activation of CCK stimulation of cofilin, as is frequently seen in other tissues (Xu et al., 2012), whereas JNK was important. CCK has also been shown to activate ROCK in pancreatic acinar cells (Sabbatini et al., 2010) and we found ROCK activation is also an important in mediating CCK’s cofilin activation. Lastly, we did not find CCK activation of PI3K was involved in cofilin activation in this cells, which is an important exception to what is frequently reported in numerous other cells with other stimuli. In pancreatic acini, activation of these signaling cascades effect on cofilin activation is principally mediated by activation of PP1/PP2A. Our results demonstrate cofilin is important in mediating pancreatic growth and enzyme secretion and that it is likely the elusive unidentified distal signaling cascade proposed in a number of older studies to mediate the effects of PP1/PP2A inhibitors stimulation on pancreatic enzyme secretion (Wagner et al., 1992; Schmidt et al., 1994). It is likely, judging by the roles of cofilin in cellular functions of other cells with other stimuli (Ohashi, 2015; Xu et al., 2021) that cofilin also plays additional roles in both other physiological (development, etc.) and pathophysiological effects in pancreatic acini (pancreatitis, pancreatic cancer growth and pathogenesis).

Furthermore, cofilin plays an important role in regulating alpha-epithelial sodium channels in collecting duct cells in the pancreas as reported in other tissues (Bukhari et al., 2020). Although our study did not examine the role of cofilin in pancreatitis, our results lead to some interesting speculation. In CCK induced pancreatitis, the high/supramaximal doses of CCK cause pancreatitis associated with the basolateral distribution of subapical F-actin, whereas physiologic CCK concentrations do not cause this (Burnham and Williams, 1982; Willemer et al., 1992; Torgerson and McNiven, 1998). The fact that our study shows that both physiologic and pathologic doses of CCK have the same effect on cofilin could be interpreted to suggest cofilin may not be involved in pancreatitis. However, with *in vivo* pancreatitis this may not be the case, and actin regulation by cofilin may still be very much involved. This could occur because at different concentrations CCK activates numerous different signaling cascades (Dufresne et al., 2006) that may be needed to interact with cofilin to produce a given effect such as pancreatitis. Therefore, cofilin at different CCK concentrations could have markedly different effects depending on other interacting cellular signal cascades.

## Data Availability

The raw data supporting the conclusion of this article will be made available by the authors, without undue reservation.
